# The network structure of mania symptoms differs between people with and without binge eating

**DOI:** 10.1111/bdi.13355

**Published:** 2023-06-12

**Authors:** Helena L. Davies, Alicia J. Peel, Jessica Mundy, Dina Monssen, Saakshi Kakar, Molly R. Davies, Brett N. Adey, Chérie Armour, Gursharan Kalsi, Yuhao Lin, Ian Marsh, Henry C. Rogers, James T. R. Walters, Moritz Herle, Kiran Glen, Chelsea Mika Malouf, Emily J. Kelly, Thalia C. Eley, Janet Treasure, Gerome Breen, Christopher Hübel

**Affiliations:** ^1^ Social, Genetic and Developmental Psychiatry (SGDP) Centre Institute of Psychiatry, Psychology, and Neuroscience, King's College London London UK; ^2^ National Institute for Health and Social Care Research (NIHR) Biomedical Research Centre, South London and Maudsley Hospital London UK; ^3^ Research Centre for Stress, Trauma and Related Conditions (STARC), School of Psychology Queen's University Belfast (QUB) Belfast, Northern Ireland UK; ^4^ Division of Psychiatry and Clinical Neurosciences, National Centre for Mental Health and MRC Centre for Neuropsychiatric Genetics and Genomics Cardiff University Cardiff UK; ^5^ Department of Biostatistics and Health Informatics King's College London London UK; ^6^ Section of Eating Disorders, Department of Psychological Medicine Institute of Psychiatry, Psychology and Neuroscience, King's College London London UK; ^7^ South London and Maudsley NHS Foundation Trust Maudsley Hospital London UK; ^8^ National Centre for Register‐based Research, Aarhus Business and Social Sciences Aarhus University Aarhus Denmark

**Keywords:** anorexia nervosa, binge‐eating disorder, bipolar disorder, bulimia nervosa, diagnosis, network analysis, signs and symptoms

## Abstract

**Objectives:**

People with bipolar disorder who also report binge eating have increased psychopathology and greater impairment than those without binge eating. Whether this co‐occurrence is related to binge eating as a symptom or presents differently across full‐syndrome eating disorders with binge eating is unclear.

**Methods:**

We first compared networks of 13 lifetime mania symptoms in 34,226 participants from the United Kingdom's National Institute for Health and Care Research BioResource with (*n* = 12,104) and without (*n* = 22,122) lifetime binge eating. Second, in the subsample with binge eating, we compared networks of mania symptoms in participants with lifetime anorexia nervosa binge‐eating/purging (*n* = 825), bulimia nervosa (*n* = 3737), and binge‐eating disorder (*n* = 3648).

**Results:**

People with binge eating endorsed every mania symptom significantly more often than those without binge eating. Within the subsample, people with bulimia nervosa most often had the highest endorsement rate of each mania symptom. We found significant differences in network parameter statistics, including network structure (*M* = 0.25, *p* = 0.001) and global strength (*S* = 1.84, *p* = 0.002) when comparing the binge eating with no binge‐eating participants. However, network structure differences were sensitive to reductions in sample size and the greater density of the latter network was explained by the large proportion of participants (34%) without mania symptoms. The structure of the anorexia nervosa binge‐eating/purging network differed from the bulimia nervosa network (*M* = 0.66, *p* = 0.001), but the result was unstable.

**Conclusions:**

Our results suggest that the presence and structure of mania symptoms may be more associated with binge eating as a symptom rather than any specific binge‐type eating disorder. Further research with larger sample sizes is required to confirm our findings.

## 1 INTRODUCTION

Eating disorders and bipolar disorder are often comorbid,^1^ which can complicate treatment.^2^ It is unclear whether this comorbidity is related to specific eating disorder symptoms or presents differently across eating disorder diagnoses.^3^ Anorexia nervosa is characterised by low weight and restrictive eating with additional symptoms of binge eating and/or purging within the subtype anorexia nervosa binge‐eating/purging.^4^ Bulimia nervosa is also characterised by binge eating and purging or other compensatory behaviours but in the absence of low weight, whilst binge eating in the absence of purging and other compensatory behaviours is classified as binge‐eating disorder.^4^

Eating disorders with binge eating (i.e. binge‐type eating disorders), in which individuals lose control and consume far greater amounts of food in a short time period than most people would,^4^ show greater comorbidity with bipolar disorder than anorexia nervosa.^1^, ^5^, ^6^, ^7^ Two per cent of people with anorexia nervosa have comorbid bipolar disorder, whereas bipolar disorder is present in 7% of people with bulimia nervosa and 9% of people with binge‐eating disorder.^6^ Individuals with comorbid bipolar disorder and binge eating report greater suicidality,^3^ have more unstable mood^3^ and emotions,^8^ and greater anxiety severity^9^ than individuals with bipolar disorder but without binge eating. The greater symptom burden explains the greater clinical impairment. Therefore, it is important to improve our understanding of bipolar disorder symptoms in individuals with and without binge eating.

A number of mechanisms have been proposed to explain this association. For instance, binge eating may alleviate both depressive^10^ and highly intense manic^11^ symptoms. Additionally, binge eating may be a side effect of mood stabilisers as these medications are associated with greater appetite and weight gain.^12^ Other possible shared underlying mechanisms for binge eating in bipolar disorder are the impulsivity^13^ and decreased ability to delay gratification^14^ observed in bipolar disorder or the higher sensitivity to rewards and poor impulse control in those at high risk of mania.^15^

One approach to increase our understanding of the relationship between eating disorder and bipolar disorder symptoms is network analysis. Networks illustrate the complex and dynamic interplay between symptoms as the development of one symptom is suggested to causally influence the development of another.^16^ Additionally, some symptoms may be of higher importance diagnostically than others.^16^ Network analysis assumes that the symptoms constitute the disorder itself, which differs from other clustering approaches, such as latent variables, which suggest that symptoms are the expression of an underlying disorder.^17^ Understanding eating disorder comorbidities via network analysis has been suggested to improve both the conceptualisation and treatment of these disorders.^18^ Networks of bipolar disorder symptoms have previously been generated^19^, ^20^ but not, to our knowledge, of bipolar disorder symptoms in people with eating disorder symptoms or diagnoses.

Bipolar disorder consists of two symptom categories, representing the extreme emotional highs (mania) and lows (depression) of mood. To maximise statistical power to detect associations^21^ and to ease the interpretability of our results, we focussed our analysis on lifetime mania symptoms. We conducted two sets of network analyses of mania symptoms to explore differences associated with binge eating and binge‐type eating disorders. In the first part of our study, we compared two mania symptom networks of individuals with and without lifetime binge eating, to understand how the presence of binge eating is associated with mania symptom structure and connectivity. At this symptom level analysis, we hypothesised that individuals with binge eating would more frequently endorse all mania symptoms. The second part of our study included a sub‐sample of the participants with binge eating who also met the criteria for a binge‐type eating disorder, and examined whether mania symptom structure and connectivity differs depending on which binge‐type eating disorder they had. We compared three mania symptom networks of individuals with (1) anorexia nervosa binge‐eating/purging subtype, (2) bulimia nervosa, and (3) binge‐eating disorder. At a disorder level, we hypothesised that people with binge‐eating disorder would endorse mania symptoms the most frequently, given that binge eating is the primary symptom in this disorder compared to bulimia nervosa, which also includes compensatory behaviours, and anorexia nervosa binge‐eating/purging, which also includes restricting and purging. Across all analyses, we hypothesised that impulsivity‐related symptoms (i.e. reckless spending, unusual and/or risky behaviour, higher libido) would be most central for groups of people with binge eating or a binge‐type eating disorder.

## 2 METHODS

### 2.1 Sample

Our sample comprised individuals from the National Institute for Health and Care Research (NIHR) BioResource cohort (*n* = 70,648) in the United Kingdom (UK). The BioResource is a collaborative resource with a databank of medical, clinical, and biological data. Participants must be 16 years and over and live in the UK. Below, we describe the sub‐cohorts of the NIHR BioResource included in our study.

#### 2.1.1 GLAD Study

The Genetic Links to Anxiety and Depression Study (GLAD; gladstudy.org.uk; *n* = 46,317) was launched in September 2018 by the NIHR. Additional details of the design and implementation of the GLAD Study are described elsewhere.^22^

#### 2.1.2 EDGI UK

The Eating Disorders Genetics Initiative (EDGI UK; edgiuk.org.uk) was launched in February 2020 by the NIHR (*n* = 6466).

Both the GLAD Study and EDGI UK are ongoing and aim to collect genetic and phenotypic data from participants with lifetime anxiety and/or depression or an eating disorder, respectively. Saliva samples for DNA have not been provided by all participants of the GLAD Study or EDGI UK and, therefore, some participants are not full members of either study. Therefore, in our study, we refer to participants from these studies as GLAD Study survey participants and EDGI UK survey participants.

#### 2.1.3 IBD BioResource

The Inflammatory Bowel Disease BioResource (IBD BioResource; www.ibdbioresource.nihr.ac.uk) was launched in January 2016 by the NIHR (*n* = 3313). The IBD BioResource is ongoing and aims to better our understanding of the role of genetics in Crohn's disease and ulcerative colitis and to improve treatment options for those suffering. Participants are recruited via participating IBD clinics across the UK.

#### 2.1.4 NHS Blood and Transplant studies

Participants are recruited from the general population to explore blood donor health. Studies include INTERVAL (*n* = 4725), COMPARE (*n* = 1956), and STRategies to Improve Donor Experiences (STRIDES; *n* = 2868).

#### 2.1.5 RTB‐GEN

The Research Tissue Bank—Generic (*n* = 5003) aims to establish a sampling framework from which people, with and without health conditions, can be selected on the basis of their genotype and/or phenotype to be invited for research studies.

For all participants other than those from EDGI UK or the GLAD Study, we obtained data only from the baseline survey within the COVID‐19 Psychiatry and Neurological Genetics (COPING) study. The COPING study was set up to monitor mental health during the pandemic via surveys sent out to participants at frequent intervals. All NIHR BioResource participants could sign up for this study throughout the pandemic. Details are described elsewhere.^23^ After completing their respective sign‐up survey, a minority of GLAD Study (*n* = 14,948) and EDGI UK (*n* = 1008) survey participants took part in the COPING study. Thus, for GLAD Study and EDGI UK survey participants, we merged their data from the relevant sign‐up survey and, if available, the COPING study baseline survey.

### 2.2 Ethics

The London—Fulham Research Ethics Committee approved the GLAD Study on 21st August 2018 (REC reference: 18/LO/1218) and EDGI UK on 29th July 2019 (REC reference: 19/LO/1254). The NIHR BioResource has been approved as a Research Tissue Bank by the East of England—Cambridge Central Committee (REC reference: 17/EE/0025). The COVID‐19 Psychiatry and Neurological Genetics study was approved by the South West—Central Bristol Research Ethics Committee on 27th April 2020 (REC reference: 20/SW/0078).

### 2.3 Measures

#### 2.3.1 Demographics

We assessed sociodemographic information including age, assigned sex at birth, years of education, height, and weight in the GLAD Study and EDGI UK sign‐up surveys and the baseline COPING study survey.

#### 2.3.2 Mania symptoms

The Mood Disorder Questionnaire (MDQ)^24^ is a screening tool for bipolar disorder. The introductory statement is ‘Has there ever been a period of time when you were not your usual self and experienced any of the following:’ and includes 13 statements assessing lifetime mania symptoms such as ‘You felt much more self‐confident than usual?’ with ‘Yes/No’ responses. A 14th item assesses the overall severity of mania symptoms. The MDQ was included in the GLAD Study sign‐up survey and the COPING study baseline survey for all participants other than GLAD Study survey participants.

#### 2.3.3 Case definition

The ED100K^25^ includes questions on lifetime eating disorder diagnosis, symptoms, cognitions, and behaviours designed to assess lifetime Diagnostic and Statistical Manual of Mental Disorders 5th edition (DSM‐5) eating‐disorder diagnostic criteria. The ED100K was optional in the GLAD Study sign‐up survey and mandatory in the EDGI UK sign‐up survey. For all sub‐cohorts other than EDGI UK, the ED100K was mandatory in the COPING study baseline survey. We first defined groups in the symptom‐level analysis using responses to the question ‘Have you ever had regular episodes of overeating or eating binges when you ate what most people would regard as an unusually large amount of food in a short period of time?’ and, to those who answered ‘Yes’, the follow‐up question of ‘When you were having regularly occurring episodes of binge eating or overeating, did you feel that your eating was out of control such that you felt you could not stop eating, or that you could not control what or how much you were eating?’. We defined individuals who answered ‘Yes’ to both questions as individuals with lifetime binge eating. Participants who answered ‘No’ to the first question form the comparison group of individuals without lifetime binge eating. We conducted a sensitivity analysis that included participants who answered ‘Yes’ to the first question but ‘No’ to the second, that is, lifetime binge eating without loss of control, which we label as ‘lifetime overeating’ (Data S1).

The Mental Health Diagnosis (MHD) questionnaire, adapted from the UK Biobank,^26^ asks participants to self‐report mental health diagnoses by a professional. The MHD was mandatory in both the GLAD Study and the EDGI UK sign‐up surveys. For all sub‐cohorts other than EDGI UK, the MHD was also mandatory as part of the COPING study baseline survey. We defined anorexia nervosa binge‐eating/purging subtype, bulimia nervosa, or binge‐eating disorder diagnosis groups. People self‐reported a diagnosis via the MHD and/or were assigned a DSM‐5^4^ algorithm‐derived diagnosis via the ED100K. To avoid overlapping cases, we applied a hierarchical categorisation commonly used in eating disorder research^27^, ^28^: anorexia nervosa binge‐eating/purging > bulimia nervosa > binge‐eating disorder. For each participant, we measured the relevant demographic and mania symptoms from the same survey in which they met the criteria for binge eating or a binge‐type eating disorder.

#### 2.3.4 Exclusion criteria

We excluded 36,422 participants with missing data on sex (*n* = 678), age (*n* = 691), mania symptoms (*n* = 5076), and/or binge eating (*n* = 30,420), leaving a total of 34,226 participants for analysis (Table 1). The absence of lifetime binge eating was determined only via the ED100K which asked about eating disorder symptoms; not having a binge‐type eating disorder, as indicated in the MHD, was not considered equivalent to having no lifetime binge eating. The ED100K was optional in the GLAD Study sign‐up survey, which strongly contributes to the high missingness of the binge–eating variable. See Table S1 for details of the differences between the included and excluded participants.

**TABLE 1:** Sub‐cohorts of the National Institute for Health and Care Research (NIHR) BioResource comprising the study sample (*n* = 34,226).

	*N*	Recruitment methods	Eligibility criteria	Recruitment area
Genetic Links to Anxiety and Depression (GLAD) Study	17,726	Social media, NHS recruitment sites	16+ years, live in the UK, lifetime experience of anxiety and/or depression	England, Wales, Scotland, Northern Ireland
Eating Disorders Genetics Initiative (EDGI UK)	1097	Social media	16+, live in England, lifetime experience of any eating disorder	England
Inflammatory Bowel Disease (IBD) cohort	2709	IBD clinics in participating hospitals across the UK	16+, have a diagnosis of Crohn's disease, ulcerative colitis, indeterminate colitis, IBD type unspecified, or suspected IBD	England, Wales, Scotland, Northern Ireland
NHS Blood and Transplant studies (COMPARE, STRIDES, INTERVAL)	8703	Blood donation centres	16+, live in England	England
Research Tissue Bank—Generic	3991	Biomedical research centres, clinical research facilities, hospital clinics, community recruitment, online	16+, live in England	England

### 2.4 Data analysis

#### 2.4.1 Descriptives

We calculated the median and interquartile ranges of continuous variables (i.e. age and body mass index) and percentage endorsement for the categorical variables for each group within the symptom‐level comparison (binge eating and no binge eating) and for each group within the diagnosis‐level comparison (anorexia nervosa binge‐eating/purging, bulimia nervosa, and binge‐eating disorder). We then performed appropriate statistical comparison tests (see Supplementary Materials section 1.2; Figures S1–S8 for details). The groups within the diagnosis‐level analysis do not sum up to the number of people with binge eating in the symptom‐level analysis because a number of participants with binge eating did not meet the diagnostic criteria for a binge‐type eating disorder (*n* = 3894).

#### 2.4.2 Network analysis

Full details of network analyses and the network comparison test are provided in Supplementary Materials section 1.3. Briefly, network analysis identifies patterns of interactivity between nodes (e.g. symptoms) in which the connections between the nodes are ‘edges’.^29^ Blue edges represent positive associations and red edges represent negative associations. We selected the 13 MDQ^24^ mania items as symptom nodes, excluding the severity item as it does not represent a symptom. To control for age and sex, we included them as nodes.

Pre‐processing checks indicated that no nodes in the symptom‐level or diagnosis‐level analysis exhibited multicollinearity as demonstrated by the *goldbricker* function from the R package *networktools*. Using the ‘nearZeroVar’ function from the R package *caret*, we established that no nodes showed near‐zero‐variance in the symptom‐level analysis. However, in the diagnosis‐level analysis, the covariate node ‘sex’ had near‐zero‐variance in the anorexia binge‐eating/purging group but not in the other two groups. We kept all nodes in all models as networks with different nodes cannot be compared.

We estimated unidirectional networks^30^ based on cross‐sectional data using the Mixed Graphic Model^31^ from the R package *mgm*. We constrained each network to the average layout to avoid misinterpretations^32^ common with the Fruchterman‐Reingold algorithm.^33^ Additionally, for each node, we identified and plotted the accuracy achieved by the marginal (i.e. unadjusted model) and the additional accuracy achieved by all nodes that are connected to that node (i.e. normalised accuracy).^34^ We identified node clusters using the walktrap algorithm.^35^ First, the likelihood of going from each node to another in a random walk is calculated (i.e. transition probability). The transition probability is the average weight of edges between two nodes and can be thought of as the ‘distance’ between two nodes. Based on these distances, clusters are formed based on the minimum possible sum of squared distances between all nodes within a cluster. Nodes are more likely to be associated with another node within the same cluster than to nodes in a different cluster.^36^ This algorithm is robust to differences in sample size^37^ and is both reliable and accurate.^36^ Age and sex were forced into their own cluster named ‘covariates’.

To generate centrality estimates, we used the *qgraph* R package. We focussed on expected influence due to established difficulties with meeting the required assumptions for betweenness and closeness in psychological networks^38^ and because expected influence takes into account more information than strength.^39^

We compared networks using the network comparison test.^40^ We used a *p*‐value threshold adjusted for the number of network comparison tests conducted in our study [*α* = (0.05/4) = 0.0125]. We generated Bonferroni‐adjusted *p*‐values for the differences in the specific edge weights and centrality of nodes across networks.

#### 2.4.3 Sensitivity analysis

We conducted sensitivity analyses to investigate the impact of the following: including participants who did not endorse any mania symptoms in the networks; unequal‐sized samples of the groups within the symptom‐level analysis compared to the groups within the diagnosis‐level analysis; the ‘loss of control’ element of binge eating; hierarchically categorising eating disorder diagnosis (i.e. we compared single eating disorder diagnosis to participants with multiple eating disorder diagnoses, labelled ‘mixed presentation’); and participants in the anorexia nervosa binge‐eating/purging group who self‐reported purging only. Full details are in the Data S1.

#### 2.4.4 Code availability

All code for this project is publicly available on Github: github.com/Helena‐D/NetworkAnalysis_symptom_vs_diagnosis.

## 3 RESULTS

### 3.1 Symptom‐level analysis

#### 3.1.1 Sample description

**FIGURE 1:**
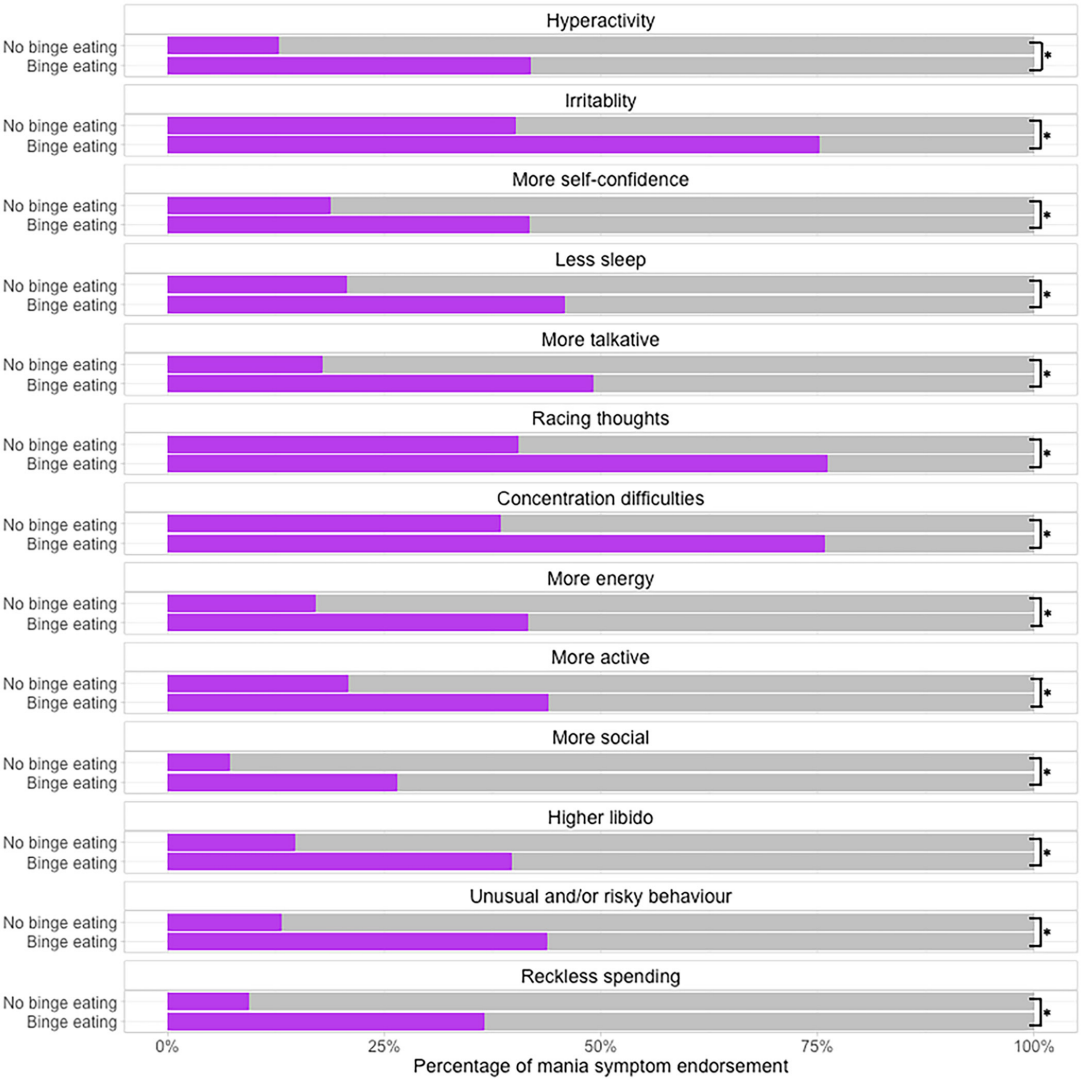
Differences in mania symptom endorsement across participants with no lifetime binge eating (*n* = 22,122) versus participants with lifetime binge eating (*n* = 12,104) from the National Institute for Health and Care Research BioResource (*n* = 34,226). Black lines indicate statistically significant differences between the groups. Further detail is in Table S2.

The groups within the symptom‐level analysis differed on a number of demographic and clinical variables (Table 2), with greater mania symptom endorsement in the binge–eating group (Figure 1; Table S2).

**TABLE 2:** Characteristics of the groups within the symptom‐level analysis from the National Institute for Health and Care Research (NIHR) BioResource (*n* = 34,226).

	No lifetime binge eating	Lifetime binge eating	Difference (*p*‐value for the significance of difference)
Total (*n*)	22,122	12,104	
Age [years] (median, IQR)	55 (23)	36 (23)	19 (2.1 × 10^−16^)
Being female	14,166 (64.0%)	10,625 (87.8%)	23.8% (2.1 × 10^−16^)
AS levels or higher	16,221 (74.6%)^a^	9370 (78.1%)^a^	3.5% (5.3 × 10^−13^)
Racially minoritised	572 (4.8%)^a^	577 (2.7%)^a^	2.1% (8.9 × 10^−24^)
Lowest lifetime BMI [kg/m^2^] (median, IQR)	23.6 (5.7)	24.2 (7.7)	0.6 (5.9 × 10^−16^)
Highest lifetime BMI [kg/m^2^] (median, IQR)	30.0 (7.9)	34.9 (13.0)	4.9 (2.1 × 10^−16^)
BMI at registration [kg/m^2^] (median, IQR)	28.2 (7.4)	31.6 (12.3)	3.4 (2.1 × 10^−16^)

*Note*: ‘Racially minoritised’ includes responses of: ‘Arab’, ‘Asian or Asian British’, ‘Black or Black British’ and ‘Mixed or multiple ethnic origins’.

Abbreviations: BMI, body mass index; FDR, false discovery rate; IQR, interquartile range.

^a^ Percentages are based on complete data, therefore may not reflect the numbers in the table. *p*‐values are FDR‐adjusted, with *α* = 0.001.

#### 3.1.2 Network stability and accuracy

**FIGURE 2:**
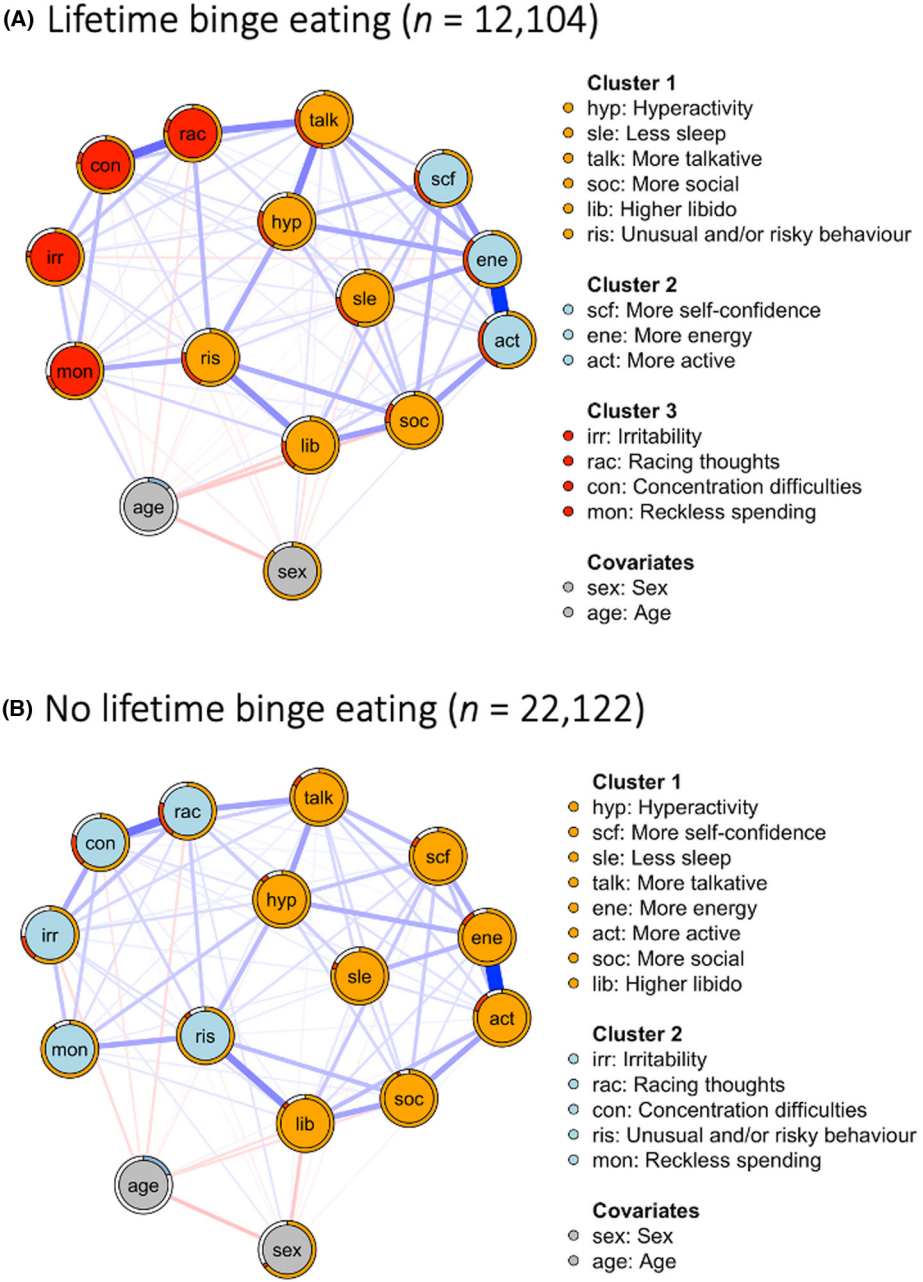
Mania symptom networks in individuals with [(A) *n* = 12,104] and without [(B); *n* = 22,122] lifetime binge eating. Blue edges indicate positive associations and red edges indicate negative associations. The width and saturation of the edge indicate the strength of the relationship, with thicker and more saturated edges representing stronger associations. Networks are plotted by calculating the average layout of the networks, and then constraining each of these networks to that layout. Within each network, the colour of the node indicates its cluster membership as defined by the walktrap algorithm (covariates have been forced into their own category). For all binary nodes, the orange colour around each node indicates the accuracy achieved by the marginal (i.e. unadjusted model); the red colour around each node indicates the additional accuracy achieved by all nodes that are connected to that node. Red + orange denotes the accuracy of the full model (i.e. marginal + additional accuracy). Normalised accuracy is depicted by the ratio of red/(red + white). Normalised accuracy is the accuracy achieved by all nodes it is connected to, beyond the accuracy achieved by the marginal. For the continuous node (i.e. age), the blue bar indicates the explained variance achieved by all nodes it is connected to.

Plots generated through the bootstrapping procedure indicated we were able to interpret differences in centrality using expected influence^30^ and that we had estimated edge weights with reasonable precision (Figures S9–S16). All bootstrapped expected influence correlation‐stability‐coefficients (CS‐coefficients) showed high stability (0.75). Figure 2 shows the mania symptom networks of those with (2a) and without (2b) lifetime binge eating.

#### 3.1.3 Clusters

We identified three clusters in the binge eating network and two clusters in the no binge eating network. ‘Irritability’, ‘reckless spending’, ‘concentration difficulties’ and ‘racing thoughts’ formed one cluster in the binge eating network. We observed a similar cluster in the no binge eating network, which also included the symptom ‘unusual and/or risky behaviour’. The rest of the symptoms formed a single cluster in the no binge eating network, but split into two in the binge eating network, including a cluster of ‘more self‐confidence’, ‘more active’ and ‘more energy’.

#### 3.1.4 Node accuracy

The normalised accuracy of each node represents the accuracy achieved by all nodes it is connected to, beyond the accuracy achieved by the marginal (i.e. unadjusted model).^34^ The nodes ‘more energy’ (0.69 and 0.51) and ‘more active’ (0.68 and 0.55) had the highest normalised accuracy in the binge‐eating network and the no binge‐eating network (Table S3).

#### 3.1.5 Centrality

**FIGURE 3:**
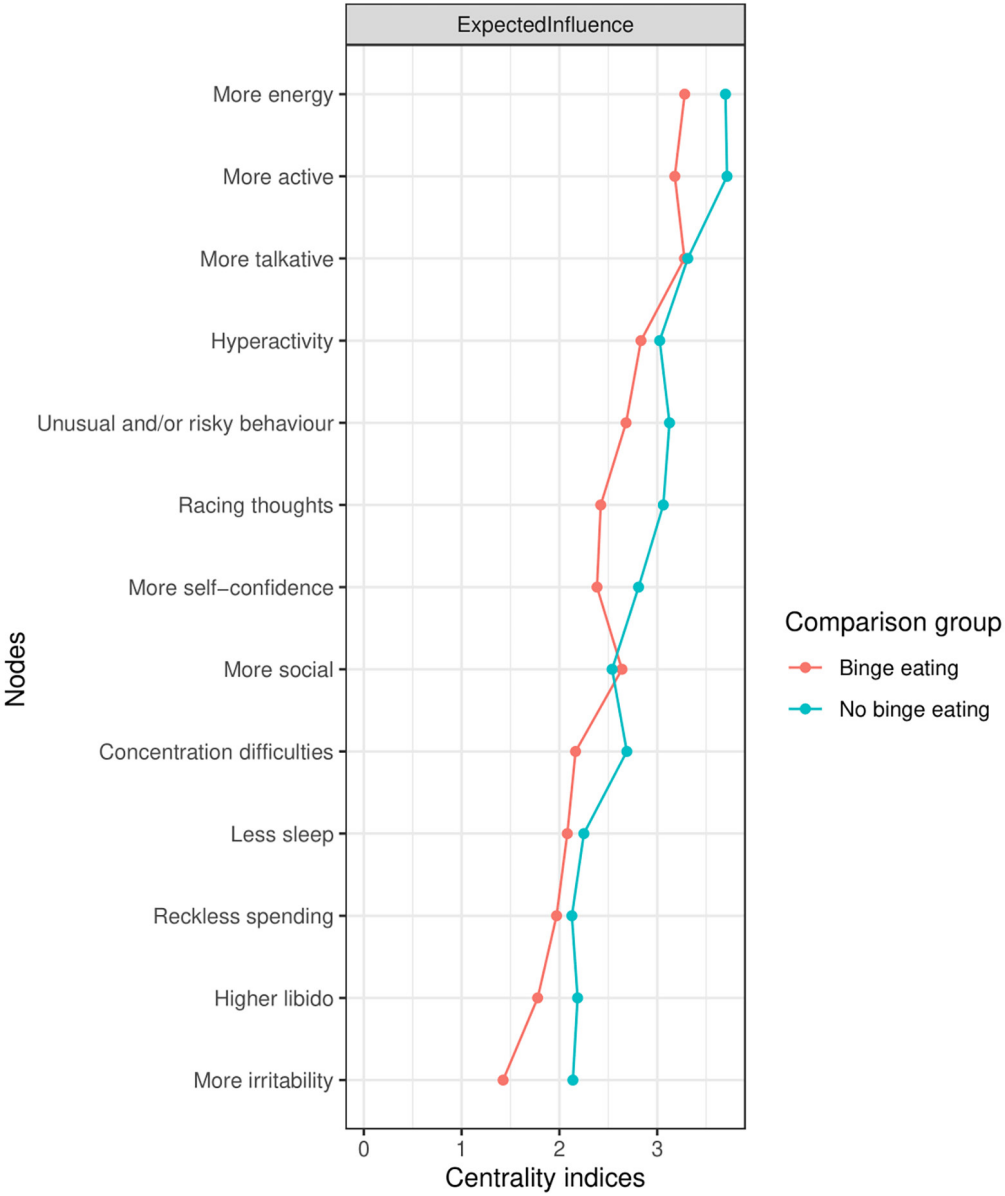
Centrality plot comparing the standardised expected influence of each symptom node in the groups within the symptom‐level analysis (lifetime binge eating *n* = 12,104; no lifetime binge eating *n* = 22,122).

‘More active’, ‘more energy’ and ‘more talkative’ were in the top three most central symptoms for both groups within the symptom‐level analysis in terms of expected influence (Figure 3). The node ‘irritability’ was the least central symptom in the binge‐eating group, and the node ‘reckless spending’ was the least central in the no binge‐eating group. A network comparison test indicated that the nodes ‘irritability’, ‘more self‐confidence’, ‘racing thoughts’, ‘concentration difficulties’, ‘more energy’, ‘more active’, ‘higher libido’ and ‘unusual and/or risky behaviour’ were significantly more central in the no binge‐eating group than the binge‐eating group (all *p*s <0.001).

#### 3.1.6 Network comparison

The network comparison test indicated that the difference in global strength (*S*) between the mania symptom network of participants with binge eating (global strength = 19.49) and participants with no binge eating (global strength = 21.33) was significant (*S* = 1.84, *p* = 0.002), with the associations in the no binge‐eating group having greater total strength. Differences in network structure (*M*) were also significant (*M* = 0.25, *p* = 0.001), indicating substantial differences in the arrangement of the symptom nodes across the two networks. We observed the strongest edge weight difference between ‘more active’ and ‘more energy’, which was 0.25 greater in the no binge‐eating network than the binge‐eating network (*p* < 0.001). Other specific differences in edge weights are described in the Data S1.

### 3.2 Diagnosis‐level analysis

#### 3.2.1 Sample description

**FIGURE 4:**
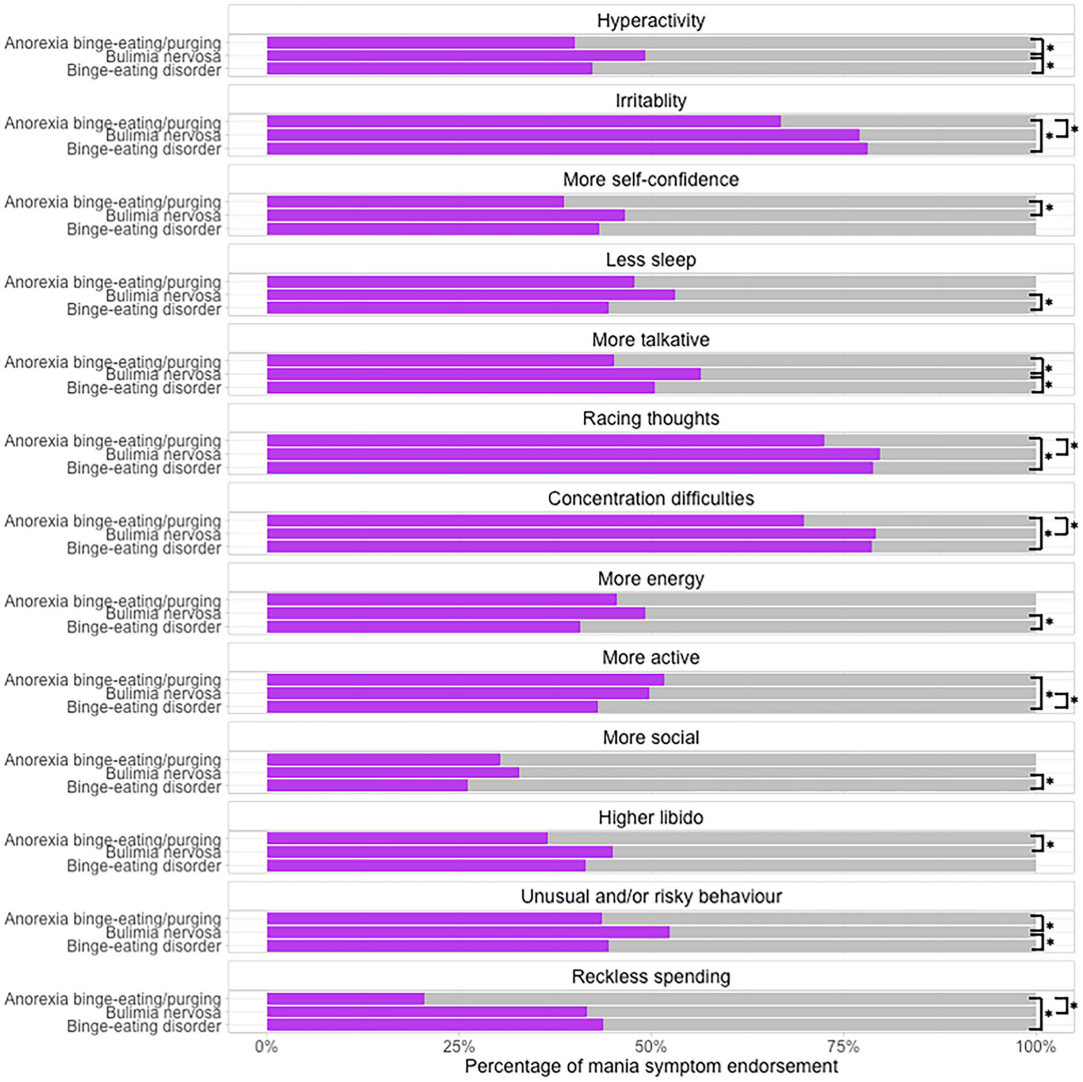
Differences in mania symptom endorsement across hierarchically categorised eating disorder groups (anorexia binge‐eating/purging *n* = 825; bulimia nervosa *n* = 3737; binge‐eating disorder *n* = 3648), from the National Institute for Health and Care Research BioResource (*n* = 8210). Black lines indicate statistically significant differences between the groups. Further detail is in Table S5.

The total number of all reported eating disorders diagnoses (i.e. self‐reported and algorithm‐derived) before the application of the pre‐defined hierarchy was *n* = 10,463 and reduced to a total of *n* = 8210 after hierarchical categorisation (Table S4). The groups within the diagnosis‐level analysis differed on a number of demographic variables (Table 3) and mania symptom endorsement (Figure 4; Table S5). For instance, participants with bulimia nervosa endorsed ‘unusual and/or risky behaviour’ significantly most often, and participants with anorexia nervosa binge‐eating/purging endorsed ‘irritability’ significantly least often.

**TABLE 3:** Characteristics of the groups within the diagnosis‐level analysis from the National Institute for Health and Care Research (NIHR) BioResource (*n* = 8210).

	ANBP	BN	BED	Significance of difference	Difference (*p*‐value for the significance of difference)		
					ANBP vs. BN	ANBP vs. BED	BN vs. BED
Total	825	3737	3648				
Age [years] (median, IQR)	26 (15)	33 (21)	37 (22)	2.5 × 10^−74^	7 (7.8 × 10^−31^)	11 (1.0 × 10^−69^)	4 (2.0 × 10^−24^)
Being female	799 (96.8%)	3505 (93.8%)	3195 (87.6%)	6.6 × 10^−28^	3.0% (7.9 × 10^−4^)	9.2% (1.9 × 10^−14^)	6.2% (1.6 × 10^−19^)
AS levels or higher	659 (79.9%)^a^	2767 (74.0%)^a^	2573 (70.5%)^a^	7.5 × 10^−7^	5.9% (3.5 × 10^−5^)	9.4% (4.6 × 10^−7^)	3.5% (0.07)
Racially minoritised	59 (7.2%)^a^	197 (5.3%)^a^	149 (4.1%)^a^	0.001	1.9% (0.04)	3.1% (7.4 × 10^−4^)	1.2% (0.03)
Lowest lifetime BMI [kg/m^2^] (median, IQR)	16.1 (2.9)	23.6 (7.1)	25.8 (7.3)	2.1 × 10^−16^	7.5 (3.0 × 10^−293^)	9.7 (1.2 × 10^−153^)	2.2 (1.63 × 10^−35^)
Highest lifetime BMI [kg/m^2^] (median, IQR)	23.6 (6.4)	34.6 (13.1)	38.4 (12.5)	2.2 × 10^−316^	11 (1.7 × 10^−188^)	14.8 (5.1 × 10^−137^)	3.8 (7.8 × 10^−41^)
BMI at registration [kg/m^2^] (median, IQR)	20.5 (5.5)	30.1 (11.7)	34.9 (11.9)	1.9 × 10^−316^	9.6 (5.1 × 10^−190^)	14.4 (5.0 × 10^−281^)	4.8 (5.1 × 10^−91^)

*Note*: Diagnoses have been assigned using self‐report (via the ED100K and/or Mental Health Diagnosis [MHD]) and/or diagnostic algorithms based on the Diagnostic and Statistical Manual of Mental Disorders, Fifth Edition (DSM‐5; via the ED100K) in either the Genetic Links to Anxiety and Depression (GLAD) Study, the Eating Disorders Genetics Initiative (EDGI UK), or the COVID‐19 Psychiatry and Neurological Genetics (COPING) study. Participants are a sub‐sample of the people who report binge eating. ‘Racially minoritised’ includes Arab, Asian or Asian British, Black or Black British, and Mixed or multiple ethnic origins.

Abbreviations: ANBP, anorexia nervosa binge‐eating/purging; BED, binge‐eating disorder; BMI, body mass index; BN, bulimia nervosa; FDR, false discovery rate; IQR, interquartile range.

^a^ Percentages are based on complete data, therefore may not reflect the numbers in the table. *p*‐value threshold for significance of difference of chi‐squared tests ($\alpha =\frac{0.05}{7}=0.007$). *p*‐values in pairwise significance of difference tests are FDR‐adjusted, with *α* = 0.001.

#### 3.2.2 Network stability and accuracy

**FIGURE 5:**
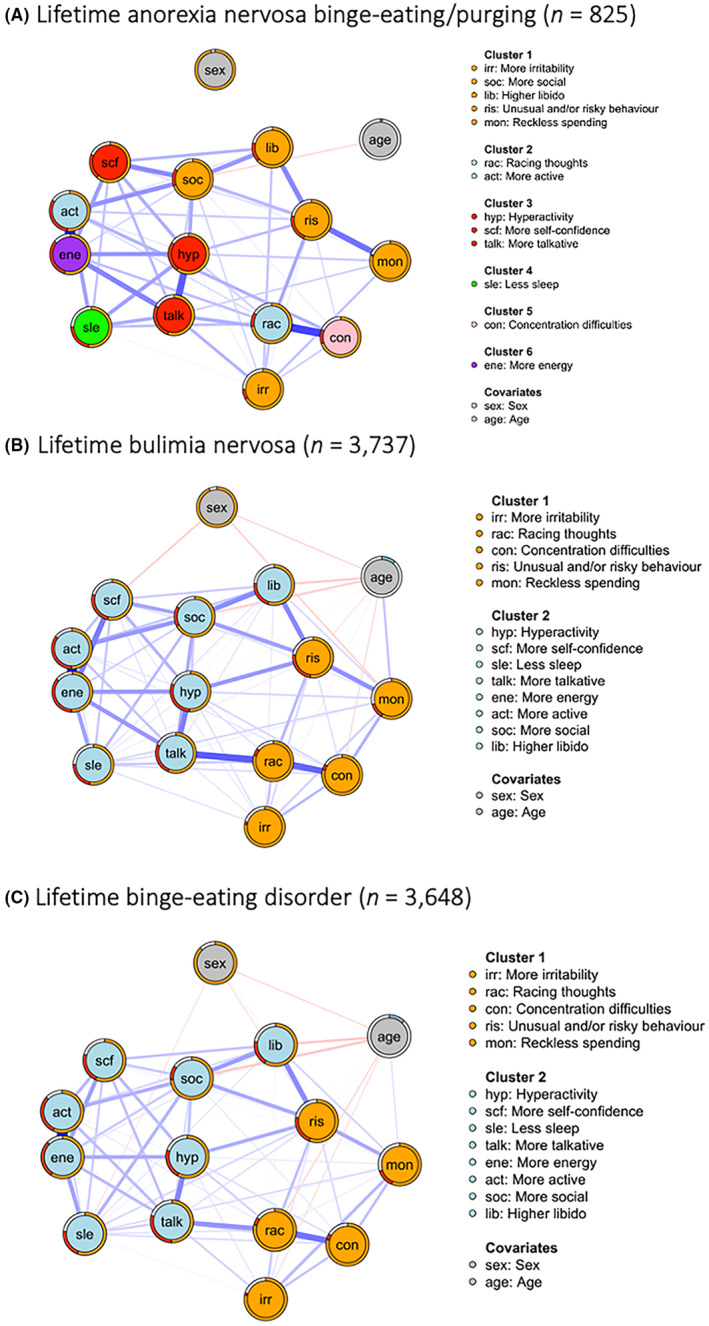
Mania symptom networks in individuals hierarchically categorised into groups of lifetime diagnosis of anorexia nervosa binge‐eating/purging [(A); *n* = 825], bulimia nervosa [(B); *n* = 3737], binge‐eating disorder [(C); *n* = 3648]. Blue edges indicate positive associations and red edges indicate negative associations. The width and saturation of the edge indicate strength of the relationship, with thicker and more saturated edges representing stronger associations. Networks are plotted by calculating the average layout of the networks, and then constraining each of these networks to that layout. Within each network, the colour of the node indicates its cluster membership as defined by the walktrap algorithm (covariates have been forced into their own category). For all binary nodes, the orange colour around each node indicates the accuracy achieved by the marginal (i.e. unadjusted model); the red colour around each node indicates the additional accuracy achieved by all nodes that are connected to that node. Red + orange denotes the accuracy of the full model (i.e. marginal + additional accuracy). Normalised accuracy is depicted by the ratio of red/(red + white). Normalised accuracy is the accuracy achieved by all nodes it is connected to, beyond the accuracy achieved by the marginal. For the continuous node (i.e. age), the blue bar indicates the explained variance by all nodes it is connected to.

All bootstrapped expected influence CS‐coefficients showed high stability (CS‐coefficients >0.75) and plots generated through the bootstrapping procedure indicated we were able to interpret differences in centrality using expected influence^30^ and that most edge weights had been estimated with reasonable precision (Figures S17–S28). However, we needed to take particular care when interpreting edge weights in the anorexia nervosa binge‐eating/purging group because estimates may be inaccurate (Figure S23), likely due to this group having the smallest sample size. Figure 5 shows the mania symptom networks of participants with lifetime anorexia nervosa binge‐eating/purging, bulimia nervosa, and binge‐eating disorder.

#### 3.2.3 Clusters

We identified the same clusters in the bulimia nervosa and binge‐eating disorder networks. One cluster was made up of ‘irritability’, ‘reckless spending’, ‘concentration difficulties’, ‘unusual and/or risky behaviour’, and ‘racing thoughts’, whilst the remaining symptoms formed the other cluster. The anorexia nervosa binge‐eating/purging network contained six clusters (not including the covariate cluster), with some clusters containing only a single symptom, such as ‘concentration difficulties’ and ‘more energy’.

#### 3.2.4 Node accuracy

The symptom ‘more energy’ (0.70–72) had the highest normalised accuracy across all three networks. ‘More active’ (0.66–70), ‘hyperactivity’ (0.53–0.62), and ‘more talkative’ (0.58–0.61) also had high normalised accuracy across all networks (Table S6).

#### 3.2.5 Centrality

‘More energy’, ‘more active’, ‘more talkative’ and ‘hyperactivity’ were the most central symptoms across all networks. ‘Irritability’ was the least central for both the binge‐eating disorder group and the bulimia nervosa group. In the anorexia nervosa binge‐eating/purging group, the node ‘reckless spending’ was the least central. Network comparison tests indicated that there were no significant differences in the expected influence of any node across all three networks (*p* > 0.8; Figure S29).

#### 3.2.6 Network comparison

We found no significant differences (*p* > 0.5) in the global strength of the mania symptom networks in participants with anorexia nervosa binge‐eating/purging (global strength = 15.10), bulimia nervosa (global strength = 17.4), or binge‐eating disorder (global strength = 17.5). Differences in network structure (*M*) were not significant in the comparison of the binge‐eating disorder group to the bulimia nervosa group (*M* = 0.21, *p* = 0.48) or to the anorexia nervosa binge‐eating/purging group (*M* = 0.45, *p* = 0.06). However, we found a significant difference in the network structure of the bulimia nervosa and anorexia nervosa binge‐eating/purging groups (*M* = 0.66, *p* = 0.001). This was due to the edge weight difference between ‘more talkative’ and ‘racing thoughts’, which was 0.66 greater in the bulimia nervosa network than the anorexia nervosa binge‐eating purging network (*p* < 0.001).

### 3.3 Sensitivity analysis

When restricting to participants who endorsed at least one mania symptom (Figures S30–S33; Tables S7 and S8), we found significant differences in the structure of the networks in the symptom‐level analysis (no binge eating *n* = 14,696; binge eating *n* = 11,411; *M* = 0.22, *p* < 0.001) and between the anorexia nervosa binge‐eating/purging network (*n* = 751) and the bulimia nervosa network (*n* = 3576) (*M* = 0.73, *p* < 0.001). Significant edge weight differences are described in the Supplementary Materials. The difference in the global strength of the networks within the symptom‐level analysis was no longer significant.

When we down‐sampled the symptom‐level analysis groups to the size of the smallest group in the diagnosis‐level analysis (Figure S34; *n* = 825), we found that global strength differed significantly (binge eating = 12.79; no binge eating = 16.57; *p* < 0.001) but not network structure. We found similar results when we down‐sampled to the size of the second smallest group in the diagnosis‐level analysis (Figure S35; *n* = 3648), with significant differences in global strength (binge eating = 17.21; no binge eating = 19.71, *p* < 0.001) but not in network structure.

#### 3.3.1 Symptom‐level‐specific sensitivity analyses

**FIGURE 6:**
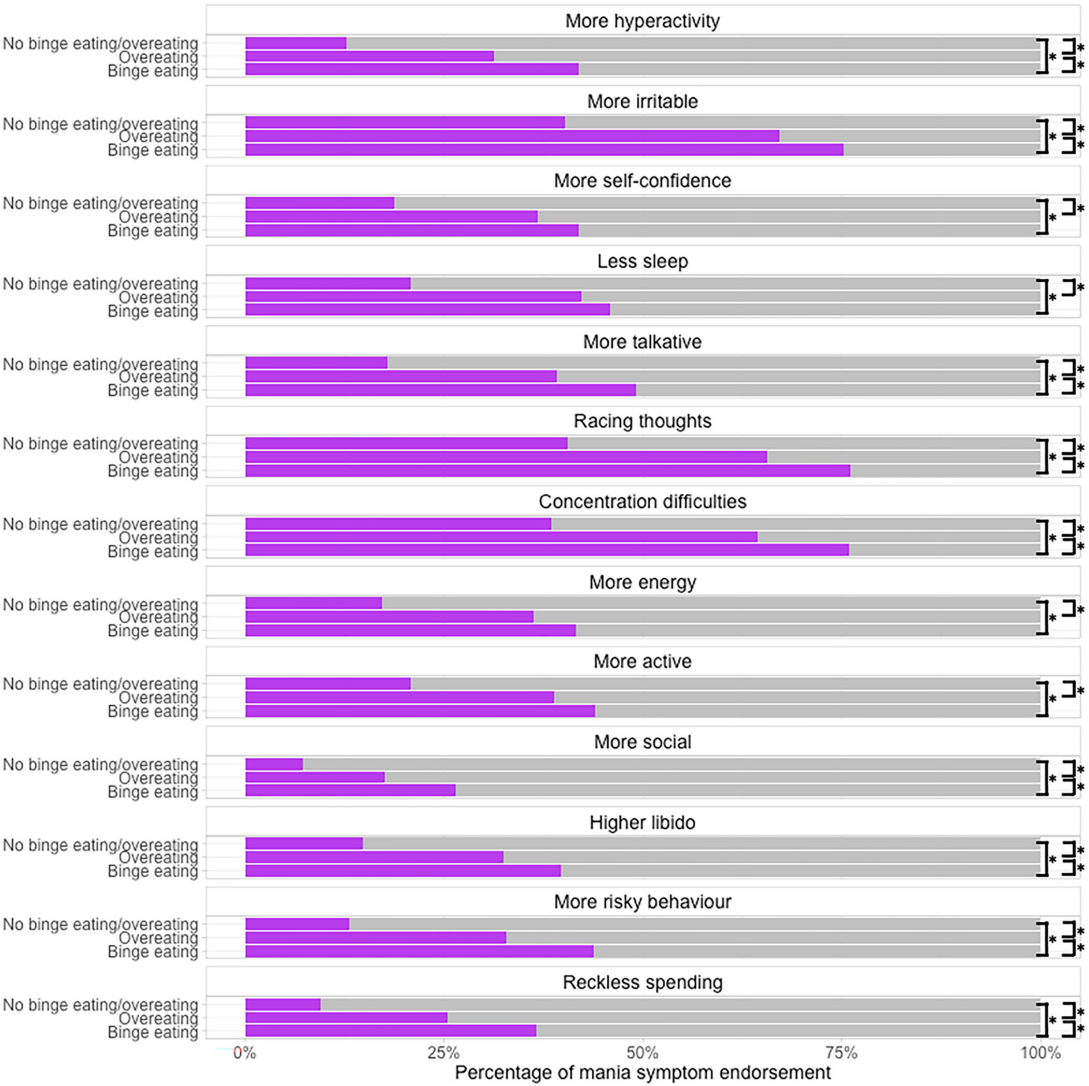
Differences in mania symptom endorsement across binge‐eating groups in a sensitivity analysis (no lifetime binge eating *n* = 22,122; lifetime overeating *n* = 926 [i.e. without loss of control]; lifetime binge eating *n* = 12,104 [i.e. with loss of control]) from the National Institute for Health and Care Research BioResource. Black lines indicate statistically significant differences between the groups.

The network of people with lifetime overeating was not significantly different to either the binge‐eating group or the no binge‐eating group (Figures S36 and S37). However, people with lifetime overeating endorsed all mania symptoms more than those without binge eating but less than those with binge eating (Figure 6; Tables S9 and S10).

#### 3.3.2 Diagnosis‐level‐specific sensitivity analyses

We found no significant differences in the networks of participants with a single diagnosis of anorexia binge‐eating purging (*n* = 445), bulimia nervosa (*n* = 1951), or binge‐eating disorder (*n* = 3648; Figures S38 and S39; Table S11). To investigate this further, we compared these networks to a network of people with a mixed presentation (*n* = 2166); the network structure of participants with a single diagnosis of anorexia nervosa binge‐eating/purging differed significantly from the network of those with a mixed presentation (M = 0.68; *p* = 0.005). Finally, we eliminated the anorexia nervosa purging‐only cases (*n* = 425; Figures S40 and S41; Table S12) and found no significant differences in the global strength of the networks within the diagnosis‐level analysis. However, the network structure of the anorexia nervosa binge‐eating network differed significantly from the bulimia nervosa network (*M* = 0.74, *p* = 0.002). The edge weight between ‘racing thoughts’ and ‘more talkative’ was significantly stronger in the bulimia nervosa network (0.74) than the anorexia binge‐eating network (0, *p* < 0.001). We describe all results in full detail in Supplementary Materials section 3.

## 4 DISCUSSION

Consistent with our hypothesis, people with binge eating self‐reported all mania symptoms significantly more often than people without binge eating. In our symptom‐level analysis, we found significant differences in network structure and global strength; the connections between the symptom nodes in the no binge‐eating group were significantly stronger than those in the binge‐eating group. However, this densely‐connected symptom pattern was explained by the large proportion of participants without binge eating who also did not endorse any mania symptoms (34%). After the removal of these participants, significant differences in network structure, but not global strength, were maintained. Nonetheless, down‐sampling resulted in a loss of these significant differences in network structure, as expected due to smaller sample sizes and less statistical power.

Further, we found some significant differences in the endorsement rates of mania symptoms across people with different binge‐type eating disorders. However, contrary to our hypothesis that people with binge‐eating disorder would have the highest endorsement rates across all mania symptoms, we found that those with bulimia nervosa most often had the highest endorsement across the groups. For example, people with bulimia nervosa endorsed ‘unusual and/or risky behaviour’, ‘hyperactivity’ and ‘more talkative’ significantly more often than both other groups. Further, they endorsed ‘more self‐confidence’ significantly more often than those with anorexia nervosa binge‐eating/purging, and ‘less sleep’ significantly more often than those with binge‐eating disorder. Given that binge eating and purging are considered impulsive behaviours,^41^ the presence of both of these behaviours in the majority of people with bulimia nervosa may explain the higher endorsement rates in this group. People with anorexia nervosa binge‐eating/purging often endorsed mania symptoms the least, with the exception of ‘more active’ which they endorsed significantly more often than those with binge‐eating disorder. High activity is common in anorexia nervosa and may in fact play a key role in its development and progression.^42^ Further, anorexia nervosa and physical activity are genetically correlated,^43^, ^44^ meaning that they have shared genetic influences. Therefore, mania items that are associated with increased physical activity may be less indicative of mania in those with anorexia nervosa because high physical activity can be a transdiagnostic symptom.

Additionally, we found no significant differences in the density or structure of the mania symptom networks of people with binge‐eating disorder versus those with bulimia nervosa or anorexia nervosa binge‐eating/purging. The network of people with anorexia nervosa binge‐eating/purging differed significantly in structure, but not density, to the bulimia nervosa group network. Specifically, the relationship between the symptom of ‘more talkative’ and ‘racing thoughts’ was significantly stronger in the bulimia nervosa network than in the anorexia nervosa binge‐eating/purging network. However, our sensitivity analyses revealed this may be driven by the presence of mixed presentations in our main analysis. Further, the edge weights in the anorexia nervosa binge‐eating/purging network were the least precise. Nonetheless, further exploration of the different association between ‘more talkative’ and ‘racing thoughts’ across these two groups is warranted.

Our results did not support our hypothesis that the impulse‐related nodes (i.e. reckless spending, unusual and/or risky behaviour, higher libido) would be the most central in the binge eating and binge‐type eating disorder networks. Whether centrality estimates are the strongest signifiers of therapeutic importance of the symptoms is debatable,^38^ and frequency of endorsement has been found to be a more consistent predictor of psychiatric disorder severity than symptom centrality.^45^ In fact, we found a distinct incongruence between the symptoms' endorsement rates and their relative centrality estimates. For example, across all the networks, ‘irritability’ was one of the most highly endorsed symptoms but one of the least central nodes. Our study thus highlights how symptoms' centrality estimates can be at odds with their frequency of endorsement, and future research using network analysis should see our findings as a caution against the interpretation of symptom importance from centrality estimates alone (this has been discussed in further detail elsewhere^38^, ^46^, ^47^, ^48^). Therefore, whether the most endorsed symptoms (i.e. ‘irritability’, ‘concentration difficulties’ and ‘racing thoughts’) or the most central symptoms (i.e. ‘more energy’, ‘more active’) are the most clinically relevant needs further investigation.

Our inclusion of the accuracy of nodes attempts to broaden the measures used in network analysis to signify node importance.^34^, ^38^ Across all binge‐type eating disorders and the binge‐eating networks, high accuracy in the symptoms ‘more active’, ‘more energy’, ‘hyperactivity’ and ‘more talkative’ was reached by other mania symptoms in the network. Our pre‐processing checks suggested that no nodes measured the same underlying construct, thus connections between these nodes and other symptoms, such as the strong relationship between ‘more active’ and ‘more energy’, indicate that these nodes likely measure different constructs that influence each other (e.g. having more energy influences activity levels). However, it is still possible that these symptoms may in fact be partial measures of the same or similar underlying constructs. If so, edge weights between the two nodes might be a result of shared variance rather than possible causal relations.^46^ Further research should explore the underlying constructs of these mania symptoms in more depth, for instance, using factor analysis.

In a sensitivity analysis, we found the ‘loss of control’ element of binge eating was not associated with changes in mania symptom structure but did explain the severity of mania symptoms. This is consistent with previous research that found 44.4% of bipolar disorder patients endorsed lifetime loss of control over eating; patients with frequent loss of control over eating had greater psychiatric and medical illness burden.^49^ Inhibitory control is impaired in those with mania symptoms^50^, ^51^ and those with binge‐type eating disorders.^52^, ^53^ Further research to establish the direction of this relationship is warranted. For instance, non‐functioning inhibitory control may drive both binge eating and mania symptoms, or mania may disturb inhibitory control which in turn leads to binge eating (or vice‐versa).

Our findings must be considered in light of limitations. First, the smaller sample size of the anorexia binge‐eating/purging group meant that edge weights in this network were the most unstable. The comparatively larger number of individuals with bulimia nervosa and binge‐eating disorder may be due, in part, to the ascertainment of the GLAD Study sample^22^ and the high comorbidity of these eating disorders with depression and anxiety.^54^ Ongoing research efforts, such as EDGI UK, may help to address this imbalance. Second, we did not include depressive symptoms because increasing the number of symptoms in a network reduces statistical power for network analyses. Future network analyses with larger sample sizes should also include depressive symptoms to cover the full range of bipolar disorder symptomatology. Third, participants in our sample were majority female and white, limiting our ability to extrapolate our results to the wider population. Fourth, compared with the UK population (~1%),^55^, ^56^ our sample contained a higher percentage of patients with IBD due to recruitment from the NIHR IBD BioResource. IBD is associated with greater psychiatric comorbidity.^57^, ^58^ However, the percentage of people with IBD was low in the binge‐type eating disorder groups (1.1%–1.5%) and the binge‐eating group (2.7%), and highest in the group without binge eating (11.4%).

To our knowledge, this is the first study to investigate differences in mania symptom networks across people with binge eating at both a symptom and diagnosis level. Overall, we can conclude from our findings that the presence and structure of mania symptoms appear to be more clearly tied to the symptom of binge eating than to any one binge‐type eating disorder. However, we had more statistical power to detect smaller differences in network structure in the larger symptom‐level groups, which were not detected when we reduced the sample size. Further, we found some evidence of network structure differences between the anorexia nervosa binge‐eating/purging group and the bulimia nervosa group. Nonetheless, we caution the interpretation of these differences due to the small sample size in the anorexia binge‐eating/purging network and thus less precise edge weight estimations as well as the instability of these findings in the sensitivity analyses. Further research with larger samples of people with binge‐type eating disorders, specifically anorexia nervosa binge‐eating/purging, is needed to confirm our findings.

## FUNDING INFORMATION

This work was supported by the National Institute for Health and Care Research (NIHR) BioResource (RG94028, RG85445), NIHR Biomedical Research Centre (IS‐BRC‐1215‐20018), HSC R&D Division, Public Health Agency (COM/5516/18), MRC Mental Health Data Pathfinder Award (MC_PC_17217), and the National Centre for Mental Health funding through Health and Care Research Wales. Prof Eley and Prof Breen are part‐funded by a program grant from the UK Medical Research Council (MR/V012878/1). Helena Davies and Alicia Peel acknowledge funding from the Economic and Social Research Council (ESRC) as part of a PhD studentship. Dr Hübel acknowledges funding from Lundbeckfonden (R276‐2018‐4581). Dr Herle is funded by a fellowship from the Medical Research Council UK (MR/T027843/1). Jessica Mundy acknowledges funding from the Lord Leverhulme Charitable Grant. Brett N. Adey acknowledges funding through a Pre‐doctoral Fellowship from the NIHR (NIHR301067).

## CONFLICT OF INTEREST STATEMENT

Prof Breen has received honoraria, research or conference grants and consulting fees from Illumina, Otsuka, and COMPASS Pathfinder Ltd. Prof Walters has received grant funding from Takeda for work unrelated to the GLAD Study. The remaining authors have nothing to disclose.

## Supporting information


Data S1.

## Data Availability

The GLAD Study, EDGI UK, and COPING study data are not publicly available however are available via a data request application to the NIHR BioResource (https://bioresource.nihr.ac.uk/using‐our‐bioresource/academic‐and‐clinical‐researchers/apply‐for‐bioresource‐data/).
