# Supplementary material for: The network structure of mania symptoms differs between people with and without binge eating
Source: Bipolar Disord. 2023 Jun 12;25(7):592–607. doi: 10.1111/bdi.13355 (PMC10768381; doi:10.1111/bdi.13355)
Supplement: Supplementary file 1 — Data S1. [file BDI-25-592-s001.docx]

**Supplementary materials**

**The network structure of mania symptoms differs between people with and without binge eating**

**Authors:** Helena L. Davies^a(0000-0002-9419-1009)^, Alicia J. Peel^a(0000-0002-6144-5412)^, Jessica Mundy^a,b(0000-0001-5513-8902)^, Dina Monssen^a,b*(0000-0003-0080-0799)^, Saakshi Kakar^a,b(0000-0003-1677-1857)^, Molly R. Davies^a,b(0000-0003-3483-9907)^, Brett N. Adey^a,b(0000-0003-4356-4079)^, Chérie Armour^c(0000-0001-7649-3874)^, Gursharan Kalsi^a,b(0000-0002-5156-7176)^, Yuhao Lin^a,b^, Ian Marsh^a,b(0000-0002-1121-9062)^, Henry C. Rogers^a,b(0000-0003-2531-7496)^, James T. R. Walters^d(0000-0002-6980-4053)^, Moritz Herle^e(0000-0003-3220-5070)^, Kiran Glen^a,b(0000-0002-2831-3266)^, Chelsea Mika Malouf^a,b(0000-0002-5564-7464)^, Emily J Kelly^a,b(0000-0002-0118-4994)^, Thalia C. Eley^a,b(0000-0001-6458-0700)^, Janet Treasure^f,g(0000-0003-0871-4596)^, Gerome Breen^a,b(0000-0003-2053-1792)^, Christopher Hübel^a,b,h(0000-0002-1267-8287)^

^a^Social, Genetic and Developmental Psychiatry (SGDP) Centre, Institute of Psychiatry, Psychology, and Neuroscience, King’s College London, London, UK

^b^National Institute for Health and Social Care Research (NIHR) Biomedical Research Centre, South London and Maudsley Hospital, London, UK

^c^Research Centre for Stress, Trauma & Related Conditions (STARC), School of Psychology, Queen’s University Belfast (QUB), Belfast, Northern Ireland, UK

^d^National Centre for Mental Health and MRC Centre for Neuropsychiatric Genetics and Genomics, Division of Psychiatry and Clinical Neurosciences, Cardiff University, Cardiff, UK

^e^Department of Biostatistics & Health Informatics, King’s College London, London, UK

^f^Department of Psychological Medicine, Section of Eating Disorders, Institute of Psychiatry, Psychology & Neuroscience, King’s College London, London, UK

^g^South London and Maudsley NHS Foundation Trust, Maudsley Hospital, Denmark Hill, London, UK

^h^National Centre for Register-based Research, Aarhus Business and Social Sciences, Aarhus University, Aarhus, Denmark

*Present address: Department of Psychological Medicine, Section of Eating Disorders, Institute of Psychiatry, Psychology & Neuroscience, King’s College London, London, UK

**1 SUPPLEMENTARY METHODS**

**1.1 Measures**

**1.1.1 ED100K**

The ED100K questionnaire begins by asking participants to report any lifetime eating disorder diagnoses, before screening participants for each eating disorder based on individual symptoms. For instance, the question '*Have you ever had a period of time when you weighed much less than other people thought you ought to weigh?*' screens participants for potential anorexia nervosa. Participants are subsequently directed to the relevant sections of the questionnaire based on their answers to the screening questions. The questionnaire contains blocks for symptoms of anorexia nervosa, atypical anorexia nervosa, and binge eating, weight loss, and compensatory behaviours. Based on combinations of these symptoms, we assigned each participant a probable diagnosis using DSM-5 algorithms.

**1.1.2 Exclusion criteria**

As shown in Supplementary Table 1, we assessed differences in basic demographics across the participants included in analysis (*n* = 34,226) versus the participants excluded from analysis (*n* = 36,422). Compared to participants included in analyses, based upon the information available for excluded participants, excluded participants were significantly younger (33 years vs. 48 years, *p* < 2.1x10^-16^) and had lower BMIs at registration (28.4 vs 29, *p* = 2.4x10^-26^), lower lifetime lowest BMIs (21.6 vs. 23.8, *p* = 1.5x10^-238^), and lower lifetime highest BMIs (29.6 vs 31.1, *p ­*= 6.7x10^-56^). More excluded participants were female (78.2% vs. 72.4%, *p* = 1.9x10^-69^) and racially minoritised (6.3% vs. 3.4%, *p* = 1.6x10^-67^), and fewer had AS levels or higher (74.3% vs 75.9%, *p* = 3.9x10^-6^) (Supplementary Table 1).

**SUPPLEMENTARY TABLE 1** Characteristics of the participants from the symptom level analysis (*n* = 34,226) versus participants excluded from analyses (*n* = 36,422) due to missing data. Participants are from the National Institute for Health and Care Research (NIHR) BioResource. ‘Included participants’ captures all participants either with lifetime binge eating (*n* = 12,104) or no lifetime binge eating (*n* = 22,122), as answered in either the Genetic Links to Anxiety and Depression (GLAD) Study, the Eating Disorders Genetics Initiative (EDGI UK), and/or the COVID-19 Psychiatry and Neurological Genetics (COPING) study.

|  | **Included participants** | **Excluded participants** | **Difference (Significance of difference test)** |
| --- | --- | --- | --- |
| Total | 34,226 | 36,422 |  |
| Age (median, IQR) | 48 (29) | 33 (23) | 15 (2.1x10^-16^) |
| Being female | 24,791 (72.4%) | 26,996 (78.2%)† | 5.8% (1.9x10^-69^) |
| AS levels or higher | 25,591 (75.9%)† | 24,612 (74.3%)† | 1.6% (3.9x10^-6^) |
| Racially minoritised | 1,149 (3.4%)† | 2,103 (6.3%)† | 2.9% (1.6x10^-67^) |
| Lowest lifetime BMI [kg/m^2^] (median, IQR) | 23.8 (8.2) | 21.6 (6.3) | 2.2 (1.5x10^-238^) |
| Highest lifetime BMI [kg/m^2^]  (median, IQR) | 31.1 (9.7) | 29.6 (11.7) | 1.5 (6.7x10^-56^) |
| BMI at registration [kg/m^2^]  (median, IQR) | 29.0 (8.9) | 28.4 (9.9) | 0.6 (2.4x10^-26^) |

Note: BMI = body mass index, IQR = interquartile range. ‘Racially minoritised’ includes: Arab, Asian or Asian British, Black or Black British, and Mixed or multiple ethnic origins. † Percentages are based on complete data, therefore may not reflect the numbers in the table. P-values are FDR-adjusted, with $\alpha$ = 0.001.

**1.2 Descriptives**

We first generated histograms and Q-Q plots to evaluate whether normality was violated (see Supplementary Figures 1-8 below), and conducted Levene’s test in R using the *car* package to evaluate homoscedasticity. For continuous variables, we conducted t-tests for the groups in the symptom-level analysis and analysis of variance (ANOVA) for the groups in the diagnosis-level analysis, or non-parametric equivalents if normality or homoscedasticity were violated. Following the ANOVA, we investigated statistically significant differences at the Bonferroni-adjusted significance using Dunn’s post-hoc tests. We judged statistical significance of the pairwise comparisons using False Discovery Rate (FDR) adjusted *p*-values with a significance threshold of 0.001. For categorical variables on both the symptom-level and diagnosis-level, we calculated chi-squared tests for comparisons. For the diagnosis-level comparisons, we further investigated differences for variables showing significance at the Bonferroni-adjusted alpha threshold, using pairwise comparison tests and judged significance via FDR-adjusted *p*-values with a significance threshold of 0.001.

**1.2.1 Assessing assumptions**

Below, we have displayed the histograms and Q-Q plots for each continuous variable within each comparison group, calculated to assess normality. All variables violated the assumption of normality and homoscedasticity, thus we used non-parametric equivalents of t-tests (Mann-Whitney) or ANOVA (Kruskal-Wallis one-way ANOVA).

**
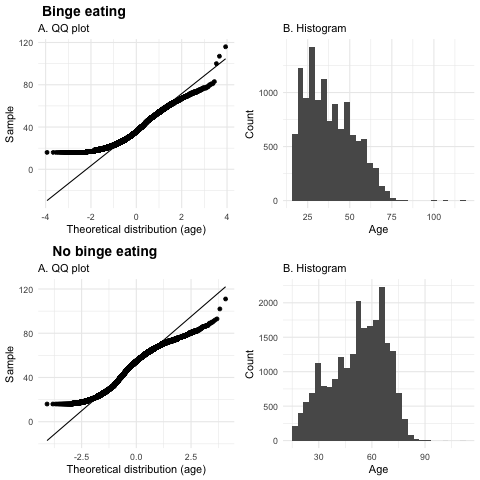
**

**SUPPLEMENTARY FIGURE 1** Histogram and Q-Q plots indicating the age distribution of participants with (*n* = 12,104) and without lifetime binge eating (*n* = 22,122).

**
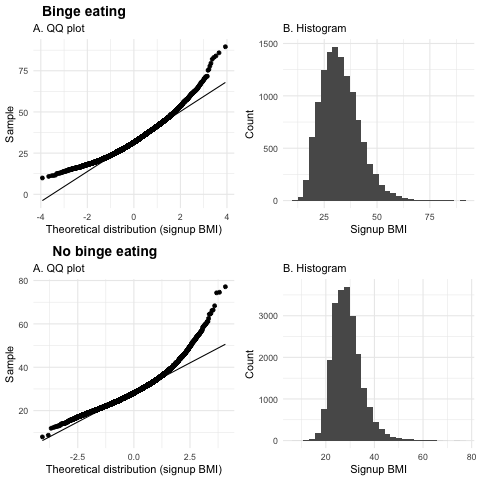
**

**SUPPLEMENTARY FIGURE 2** Histogram and Q-Q plots indicating the BMI at registration distribution of participants with (*n* = 12,104) and without lifetime binge eating (*n* = 22,122).

**
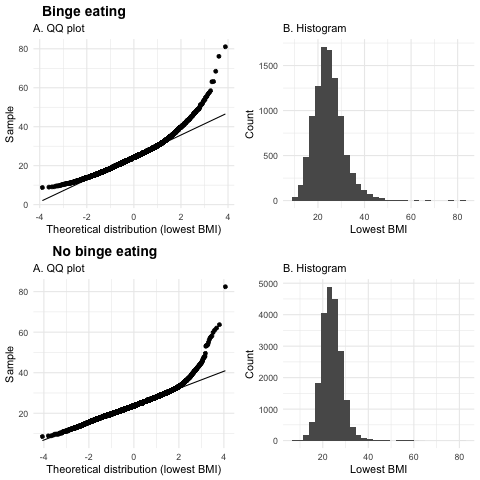
**

**SUPPLEMENTARY FIGURE 3** Histogram and Q-Q plots indicating the lifetime lowest BMI distribution of participants with (*n* = 12,104) and without lifetime binge eating (*n* = 22,122).

**
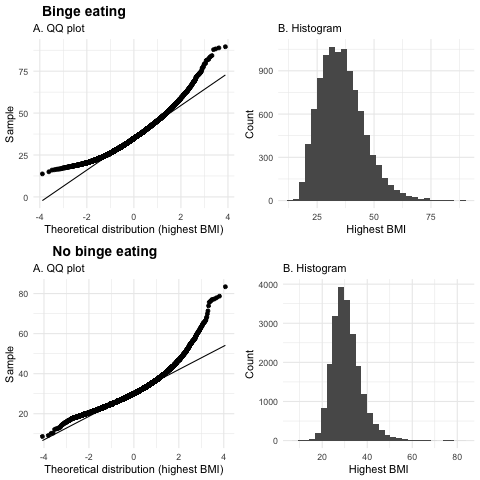
**

**SUPPLEMENTARY FIGURE 4** Histogram and Q-Q plots indicating the lifetime highest BMI distribution of participants with (*n* = 12,104) and without lifetime binge eating (*n* = 22,122).


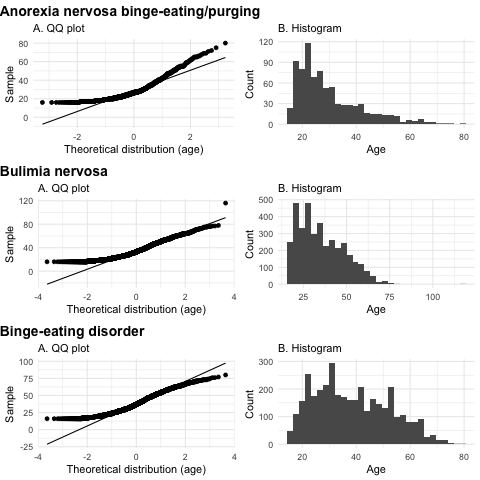


**SUPPLEMENTARY FIGURE 5** Histogram and Q-Q plots indicating the age distribution of participants with anorexia nervosa binge-eating/purging (*n* = 825), bulimia nervosa (*n* = 3,737), and binge-eating disorder (*n* =3,648).

**
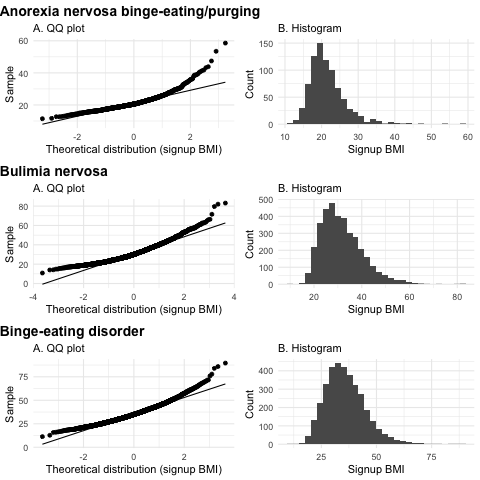
**

**SUPPLEMENTARY FIGURE 6** Histogram and Q-Q plots indicating the BMI at registration distribution of participants with anorexia nervosa binge-eating/purging (*n* = 825), bulimia nervosa (*n* = 3,737), and binge-eating disorder (*n* =3,648).


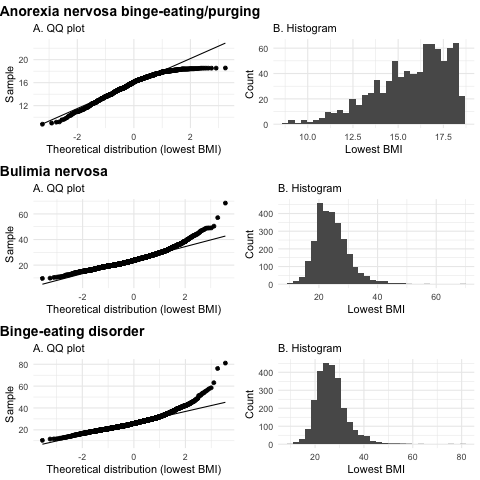


**SUPPLEMENTARY FIGURE 7** Histogram and Q-Q plots indicating the lifetime lowest BMI distribution of participants with anorexia nervosa binge-eating/purging (*n* = 825), bulimia nervosa (*n* = 3,737), and binge-eating disorder (*n* = 3,648).

**
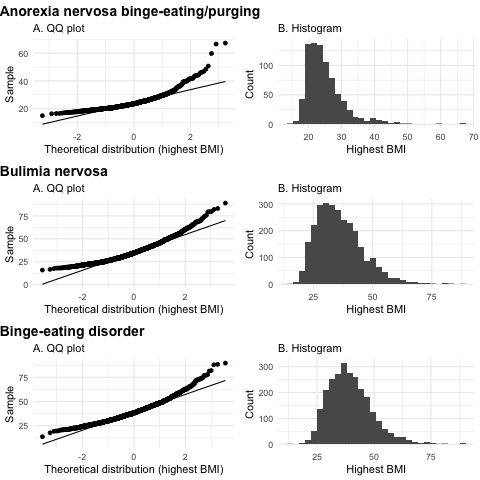
**

**SUPPLEMENTARY FIGURE 8** Histogram and Q-Q plots indicating the lifetime highest BMI distribution of participants with anorexia nervosa binge-eating/purging (*n* = 825), bulimia nervosa (*n* = 3,737), and binge-eating disorder (*n* = 3,648).

**1.3 Network analysis**

**1.3.1 Node selection**

We selected the 13 Mood Disorder Questionnaire (MDQ)[^1^](https://paperpile.com/c/sZAmz0/gd6Yp) mania items as symptom nodes, excluding the severity item as it does not represent a symptom.

**1.3.2 Network estimation**

We estimated unidirectional networks[^2^](https://paperpile.com/c/sZAmz0/Su8nU) based on cross-sectional data using the Mixed Graphic Model[^3^](https://paperpile.com/c/sZAmz0/qOU1d) from the R package *mgm.* Appropriate regressions to estimate symptom-symptom associations were automatically performed in R. To reduce false positives and circumvent the issue of multiple testing, we utilised Lasso regularisation to shrink small edge weights to zero[^4,5^](https://paperpile.com/c/sZAmz0/NZUfj+lpjtv). We used a hyperparameter (gamma) of 0.25 to adjust model sparsity and generate weighted adjacency matrices. To visualise the networks, we used the *qgraph* R package.

**1.3.3 Accuracy and stability**

We used the *bootnet* package to assess network accuracy and stability. To assess edge weight accuracy, we bootstrapped 95% confidence intervals of the edges by resampling participants with replacement and plotting the range[^2^](https://paperpile.com/c/sZAmz0/Su8nU). To assess centrality stability, we re-estimated each network 1,000 times iteratively with smaller sub-samples (i.e., case-dropping bootstraps)[^2^](https://paperpile.com/c/sZAmz0/Su8nU). We estimated the correlation between the original centrality estimates (specifically expected influence) and each newly-generated centrality estimate and generated the correlation stability coefficient (CS-coefficient). CS-coefficients of above 0.5 are acceptable[^2^](https://paperpile.com/c/sZAmz0/Su8nU).

**1.3.4 Bootstrapped difference test within networks**

To explore whether significant differences in edge weights or node centrality estimates existed within each network, we conducted difference tests of pairs of edge weights or centrality estimates using bootstrapped values from the network accuracy assessments[^2^](https://paperpile.com/c/sZAmz0/Su8nU). These tests do not correct for multiple testing, thus caution should be taken when interpreting results. However, both tests have a type 1 error rate at least as conservative as significance level testing (α = 0.05)[^2^](https://paperpile.com/c/sZAmz0/Su8nU).

**1.3.5 Centrality estimates**

Node centrality assesses symptom importance using multiple metrics[^6^](https://paperpile.com/c/sZAmz0/CODTP): 1) strength, the absolute sum of all edge weights linked to the node, 2) closeness, the inverse of the sum of the shortest path lengths that need to be crossed to get from said node to all other nodes, 3) betweenness, the number of shortest path lengths that the node is connected to, and 4) expected influence, the sum of all edge weights linked to the node. To generate centrality estimates, we used the *qgraph* R package. As stated in the main manuscript, we focussed on expected influence due to established difficulties with meeting the required assumptions for betweenness and closeness in psychological networks[^7^](https://paperpile.com/c/sZAmz0/WcFiG) and because expected influence takes into account more information than strength[^8^](https://paperpile.com/c/sZAmz0/xKnh3).

**1.3.6 Network comparison**

The Network Comparison Test (NCT) explores statistically significant differences across networks[^5^](https://paperpile.com/c/sZAmz0/lpjtv). The NCT permutes the networks 1,000 times, each with different subpopulations, and then compares the two networks across three measures[^5^](https://paperpile.com/c/sZAmz0/lpjtv), 1) network structure (compares edge weight distribution), 2) global strength (compares overall network connectivity), and 3) specific edge weights (evaluates which specific edge weights differ between networks, and is only tested if significant differences in network structure are found). Permutation testing, in which group membership (i.e., presence of binge eating or binge-type eating disorder status) is randomly re-assigned, helps to overcome issues with differences in sample size within comparisons. Age and sex were not included in the network comparison test, as these nodes were covariates and not symptoms.

**1.4 Sensitivity analysis**

We conducted a range of sensitivity analyses. To evaluate the impact of including participants who did not endorse any mania symptoms across all networks, we restricted our analyses to those who reported at least one mania symptom (no binge eating *n* = 14,696; binge eating *n* = 11,411; anorexia nervosa binge-eating/purging *n* = 751; bulimia nervosa *n* = 3,576; binge-eating disorder *n* = 3,488). Further, to assess the influence of unequal sized samples of the groups in the symptom-level analysis versus the groups in the diagnosis-level analysis, we down-sampled the groups in the symptom-level analysis to 825 (i.e., the size of the anorexia nervosa binge-eating/purging group) and then to 3,648 (i.e., the size of the binge-eating disorder group). Different sample sizes between the groups in the symptom-level analysis and between the groups in the diagnosis-level analysis were taken into account when we conducted the permutation tests.

**1.4.1 Symptom-level-specific sensitivity analyses**

Some of our sensitivity analyses were specific to the symptom-level analysis. To assess the importance of the ‘loss of control’ aspect of the binge eating phenotype, we constructed a network for those who reported binge eating with no loss of control, i.e., overeating (*n* = 926). These participants answered *‘Yes’* to the question *'Have you ever had regular episodes of overeating or eating binges when you ate what most people would regard as an unusually large amount of food in a short period of time?*' but answered *‘No’* to the follow-up question of *'When you were having regularly occurring episodes of binge eating or overeating, did you feel that your eating was out of control such that you felt you could not stop eating, or that you could not control what or how much you were eating?*'. We compared this network to the other networks in the symptom-level analysis (i.e., binge eating [with loss of control] and no binge eating/overeating).

**1.4.2 Diagnosis-level-specific sensitivity analyses**

Other sensitivity analyses were specific to our diagnosis-level analysis. To investigate the impact of hierarchically categorising eating disorder diagnosis, we altered the diagnostic groups to include those who had self-reported and/or reached diagnostic criteria for only one of the binge-type eating disorders (anorexia nervosa binge-eating/purging only *n* = 445, bulimia nervosa only *n* = 1,951, binge-eating disorder only *n* = 3,648). We also estimated a network for the remaining participants, who had self-reported and/or reached diagnostic criteria for more than one binge-type eating disorder, i.e., had a ‘mixed presentation’ (*n* = 2,166). Finally, we excluded cases of anorexia nervosa purging-only within the anorexia nervosa binge-eating/purging group, and re-ran our comparisons within the diagnosis-level analysis to explore comparisons of cases with binge eating only (anorexia nervosa binge-eating *n* = 400, bulimia nervosa *n* = 3,830, and binge-eating disorder 3,667). The increase in cases in the bulimia nervosa (*n* = 93) and the binge-eating disorder (*n* = 13) is due to the recategorisation of participants who were previously categorised as anorexia nervosa binge-eating purging but were then excluded from this group because they only purged at low weight.

**2 RESULTS: MAIN ANALYSES**

**2.1 Symptom-level analysis**

**SUPPLEMENTARY TABLE 2** Mania symptom endorsement of the groups within the symptom-level analysis from the National Institute for Health and Care Research (NIHR) BioResource (*n* = 34,226). Participants indicated the presence or absence of lifetime binge eating with loss of control in either the Genetic Links to Anxiety and Depression (GLAD) Study, the Eating Disorders Genetics Initiative (EDGI UK), and/or the COVID-19 Psychiatry and Neurological Genetics (COPING) study.

|  | **No binge eating** | **Binge eating** | **Difference (Significance of difference test)** |
| --- | --- | --- | --- |
| Total | 22,122 | 12,104 |  |
| Reckless spending | 2,078 (9.4%) | 4,428 (36.6%) | 27.2% (2.1x10^-16^) |
| Unusual and/or risky behaviour | 2,901 (13.1%) | 5,304 (43.8%) | 30.7% (2.1x10^-16^) |
| Higher libido | 3,272 (14.8%) | 4,797 (39.6%) | 24.8% (2.1x10^-16^) |
| More social | 1,601 (7.2%) | 3,200 (26.4%) | 19.2% (2.1x10^-16^) |
| More active | 4,609 (20.8%) | 5,324 (44.0%) | 23.2% (2.1x10^-16^) |
| More energy | 3,786 (17.1%) | 5,033 (41.6%) | 24.5% (2.1x10^-16^) |
| Concentration difficulties | 8,503 (38.4%) | 9,185 (75.9%) | 37.4% (2.1x10^-16^) |
| Racing thoughts | 8,969 (40.5%) | 9,216 (76.1%) | 35.6% (2.1x10^-16^) |
| More talkative | 3,949 (17.9%) | 5,941 (49.1%) | 31.2% (2.1x10^-16^) |
| Less sleep | 4,589 (20.7%) | 5,554 (45.9%) | 25.1% (2.1x10^-16^) |
| Irritability | 8,887 (40.2%) | 9,097 (75.2%) | 35.0% (2.1x10^-16^) |
| Hyperactivity | 2,823 (12.8%) | 5,069 (41.9%) | 29.1% (2.1x10^-16^) |
| More self-confidence | 4,158 (18.8%) | 5,062 (41.8%) | 23.0% (2.1x10^-16^) |

Note. P-values are FDR-adjusted, with $\alpha$ = 0.001.

**2.1.1 Edge weight and centrality difference tests**

Below are the bootstrapped edge weight difference test results and the bootstrapped difference tests of expected influence within the binge eating group and the no binge eating group. Bootstrapped difference tests of non-zero edge weights indicated that, out of the maximum 105 edge weights, 84 were estimated to be non-zero in the binge eating group whilst 90 were estimated as non-zero in the no binge eating group. This probably reflects the difference in sample size.


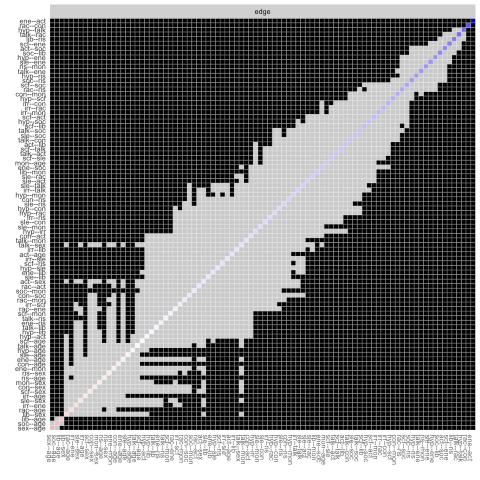


**SUPPLEMENTARY FIGURE 9** Bootstrapped non-zero edge weight difference tests in the binge eating group (*n* = 12,104). Black boxes indicate a significant difference between edge weights. Edge weights are arranged in order of strength, with the strongest edge weight on the far right of the axis. The diagonal indicates the relative strength of each edge weight, with a darker colour indicating a stronger edge weight. The colour of the diagonal corresponds to the colour of the edge weights in the network plot. Blue indicates a positive association, and red indicates a negative association. Note: mon = reckless spending, irr = irritability, con = concentration difficulties, sle = less sleep, lib = higher libido, scf = more self-confidence, rac = racing thoughts, talk = more talkative, soc = more social, ris = unusual and/or risky behaviour, ene = more energy, act = more active, hyp = hyperactivity.


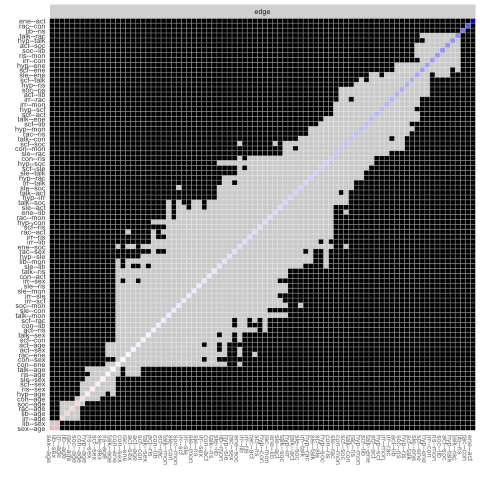


**SUPPLEMENTARY FIGURE 10** Bootstrapped non-zero edge weight difference tests in the no binge eating group (*n* = 22,122). Black boxes indicate a significant difference between edge weights. Edge weights are arranged in order of strength, with the strongest edge weight on the far right of the axis. The diagonal indicates the relative strength of each edge weight, with a darker colour indicating a stronger edge weight. The colour of the diagonal corresponds to the colour of the edge weights in the network plot. Blue indicates a positive association, and red indicates a negative association. Note: mon = reckless spending, irr = irritability, con = concentration difficulties, sle = less sleep, lib = higher libido, scf = more self-confidence, rac = racing thoughts, talk = more talkative, soc = more social, ris = unusual and/or risky behaviour, ene = more energy, act = more active, hyp = hyperactivity.


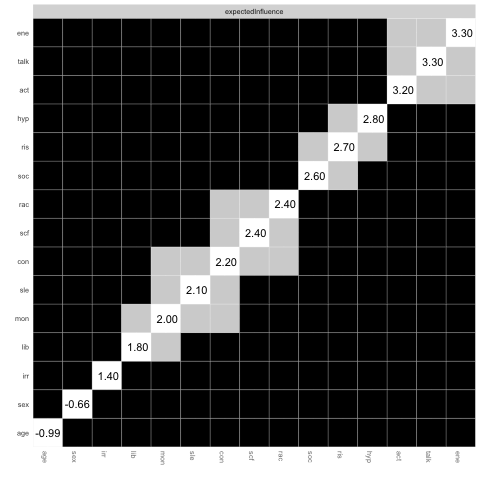


**SUPPLEMENTARY FIGURE 11** Bootstrapped difference tests of the expected influence of nodes (i.e., sum of edge weights) in the binge eating group (*n* = 12,104). Black boxes indicate a significant difference between nodes’ centrality estimates. Nodes are arranged in order of centrality, with the most central node on the far right of the axis. Note: mon = reckless spending, irr = irritability, con = concentration difficulties, sle = less sleep, lib = higher libido, scf = more self-confidence, rac = racing thoughts, talk = more talkative, soc = more social, ris = unusual and/or risky behaviour, ene = more energy, act = more active, hyp = hyperactivity.


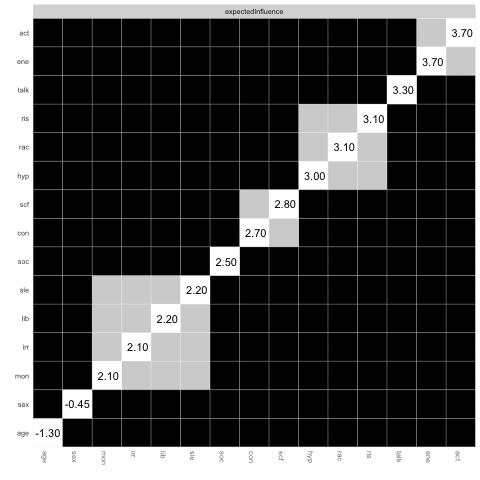


**SUPPLEMENTARY FIGURE 12** Bootstrapped difference tests of the expected influence of nodes (i.e., sum of edge weights) in the no binge eating group (*n* = 22,122). Black boxes indicate a significant difference between nodes’ centrality estimates. Nodes are arranged in order of centrality, with the most central node on the far right of the axis. Note: mon = reckless spending, irr = irritability, con = concentration difficulties, sle = less sleep, lib = higher libido, scf = more self-confidence, rac = racing thoughts, talk = more talkative, soc = more social, ris = unusual and/or risky behaviour, ene = more energy, act = more active, hyp = hyperactivity.

**Network accuracy and stability.** We generated plots via the bootstrapping procedure to evaluate edge weight accuracy and centrality stability. Below are expected influence centrality stability plots and edge weight accuracy plots within the binge eating and no binge eating group generated through the bootstrapping procedure.


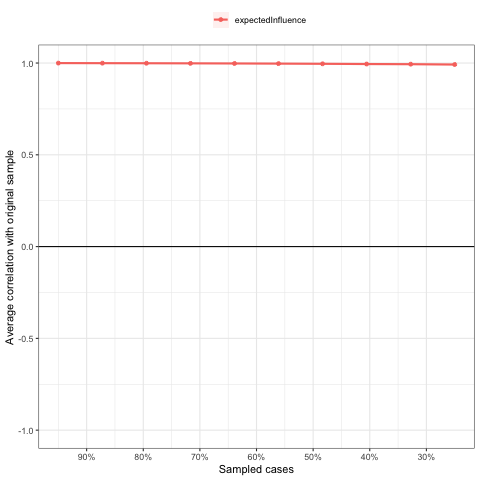


**SUPPLEMENTARY FIGURE 13** Centrality stability plot in the binge eating group (*n* = 12,104). Correlation between original order of expected influence with order of expected influence after case-dropping bootstrapping (i.e., dropping rows from the data).

**
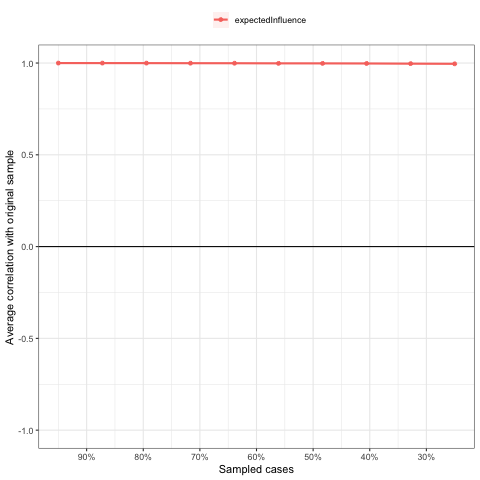
**

**SUPPLEMENTARY FIGURE 14** Centrality stability plot in the no binge eating group (*n* = 22,122). Correlation between original order of expected influence with order of expected influence after case-dropping bootstrapping (i.e., dropping rows from the data).


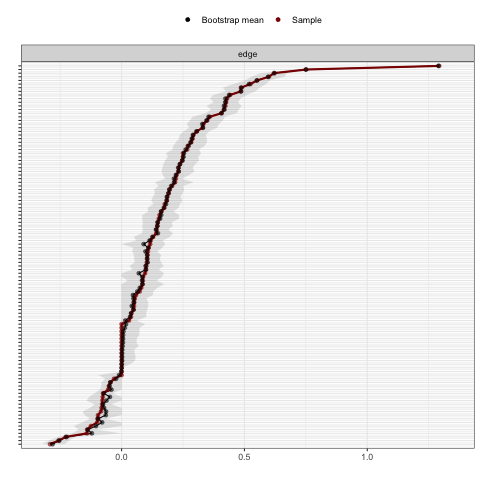


**SUPPLEMENTARY FIGURE 15** The 95% bootstrapped confidence intervals of all non-zero edge weights in the binge eating group (n = 12,104). The Y axis represents the edge weight relationships (labels removed to avoid cluttering), with each line indicating a relationship between two edges, ordered from the lowest edge weight to the highest in ascending order. The X axis represents the weight of each relationship between two edges. Sample values are demonstrated by the red line, and the black line indicates the mean of the bootstrapped estimates.


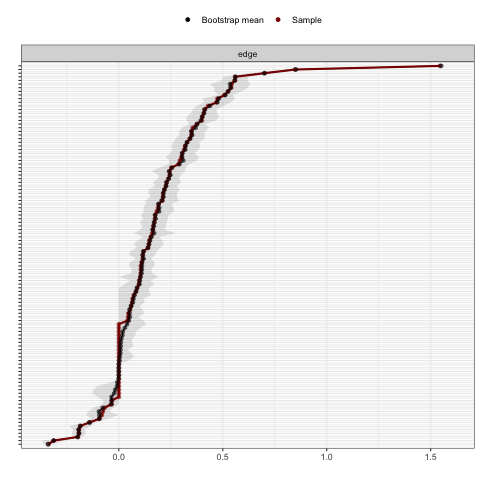


**SUPPLEMENTARY FIGURE 16** The 95% bootstrapped confidence intervals of all non-zero edge weights in the no binge eating group (*n* = 22,122). The Y axis represents the edge weight relationships (labels removed to avoid cluttering), with each line indicating a relationship between two edges, ordered from the lowest edge weight to the highest in ascending order. The X axis represents the weight of each relationship between two edges. Sample values are demonstrated by the red line, and the black line indicates the mean of the bootstrapped estimates.

**2.1.2 Network connectivity**

Bootstrapped difference tests within networks revealed that, in both networks, the strongest edges were between 'more energy' and 'more active' (edge weights = 1.29-1.54) and 'racing thoughts' and 'concentration difficulties' (edge weights = 0.75-0.85). As stated in the main manuscript, we found a significant difference in network structure between these two networks: the edge weights between: 'irritability' and 'concentration difficulties', 'more self-confidence' and 'more talkative', 'more energy' and 'more active', and 'higher libido' and 'unusual and/or risky behaviour' were significantly stronger in the no binge eating network than in the binge eating network (all *p*s < 0.001).

**2.1.3 Node accuracy.**

Supplementary Table 3 below shows the normalised accuracy[^9^](https://paperpile.com/c/sZAmz0/Nt6I) of each node in the networks within the symptom-level analysis.

**SUPPLEMENTARY TABLE 3** Normalised accuracy of each node in the binge eating group (*n* = 12,104) and the no binge eating group (*n* = 22,122). National Institute for Health and Care Research (NIHR) BioResource participants (*n* = 34,226) indicated the presence or absence of lifetime binge eating with loss of control in either the Genetic Links to Anxiety and Depression (GLAD) Study, the Eating Disorders Genetics Initiative (EDGI UK), and/or the COVID-19 Psychiatry and Neurological Genetics (COPING) study.

|  | **Normalised accuracy** | |
| --- | --- | --- |
|  | **Binge eating** | **No binge eating** |
| Hyperactivity | 0.55 | 0.31 |
| Irritability | 0.13 | 0.35 |
| More self-confidence | 0.51 | 0.30 |
| Less sleep | 0.46 | 0.18 |
| More talkative | 0.60 | 0.35 |
| Racing thoughts | 0.31 | 0.50 |
| Concentration difficulties | 0.27 | 0.46 |
| More energy | 0.69 | 0.51 |
| More active | 0.68 | 0.55 |
| More social | 0.43 | 0.22 |
| Higher libido | 0.43 | 0.22 |
| Unusual and/or risky behaviour | 0.50 | 0.28 |
| Reckless spending | 0.23 | 0.37 |

**2.2 Diagnosis-level analysis**

**2.2.1 Hierarchical categorisation**

As shown in Supplementary Table 4, the number of participants reduced from 10,463 to 8,210 following hierarchical categorisation, indicating that up to 2,253 participants had more than one binge-type eating disorder diagnosis (i.e., mixed presentation).

**SUPPLEMENTARY TABLE 4** Self-reported eating disorder diagnosis versus Diagnostic and Statistical Manual of Mental Disorders, Fifth Edition, (DSM-5) algorithm-derived diagnosis of anorexia nervosa binge-eating/purging (ANBP), bulimia nervosa (BN), and binge-eating disorder (BED) in National Institute for Health and Care Research (NIHR) BioResource participants (*n* = 8,210) before hierarchical categorisation, and number of total eating disorder diagnoses after hierarchical categorisation (ANBP > BN > BED). Algorithms used questions from the ED100K and were based on DSM-5 criteria. Self-report cases were obtained from the Mental Health Disorder (MHD) questionnaire. Responses are from the Genetic Links to Anxiety and Depression (GLAD) Study, the Eating Disorders Genetics Initiative (EDGI UK), and/or the COVID-19 Psychiatry and Neurological Genetics (COPING) study.

|  | **Anorexia nervosa binge-eating/purging** | **Bulimia nervosa** | **Binge-eating disorder** |
| --- | --- | --- | --- |
| ***Before hierarchical categorisation*** |  |  |  |
| Self-report & algorithm-derived | NA† | 628 | 1,180 |
| Algorithm-derived only | 825 | 2,156 | 2,426 |
| Self-report only | NA† | 1,291 | 1,957 |
| Total | 825 | 4,075 | 5,563 |
| ***After hierarchical categorisation*** |  |  |  |
| Total | 825 | 3,737 | 3,648 |

​​Note: † Participants were not given the option to self-report a diagnosis of anorexia nervosa binge-eating/purging. ANBP = anorexia nervosa binge-eating/purging, BN = bulimia nervosa, BED = binge-eating disorder.

**SUPPLEMENTARY TABLE 5** Mania symptom endorsement of the groups within the diagnosis-level analysis from the National Institute for Health and Care Research (NIHR) BioResource (*n* = 8,210). Diagnoses have been assigned using self-report (via the Mental Health Diagnosis [MHD] questionnaire) and/or diagnostic algorithms based on the Diagnostic and Statistical Manual of Mental Disorders, Fifth Edition (DSM-5; via the ED100K) in either the Genetic Links to Anxiety and Depression (GLAD) Study, the Eating Disorders Genetics Initiative (EDGI UK), and/or the COVID-19 Psychiatry and Neurological Genetics (COPING) study. Participants are a sub-sample of the people who report binge eating.

|  |  |  |  |  | **Difference**  **(Pairwise significance of difference)** | | |
| --- | --- | --- | --- | --- | --- | --- | --- |
|  | **ANBP** | **BN** | **BED** | **Significance of difference** | **ANBP vs. BN** | **ANBP vs. BED** | **BN vs. BED** |
| Total | 825 | 3,737 | 3,648 |  |  |  |  |
| Reckless spending | 169  (20.5%) | 1,556 (41.6%) | 1,595 (43.7%) | 2.3x10^-34^ | 21.1%  (1.9x10^-29^) | 23.2%  (2.9x10^-34^) | 2.1%  (0.07) |
| Unusual and/or risky behaviour | 360 (43.6%) | 1,956 (52.3%) | 1,622 (44.5%) | 3.4x10^-12^ | 8.7%  (1.1x10^-5^) | 0.9%  (0.7) | 7.8%  (4.4x10^-11^) |
| Higher libido | 301 (36.5%) | 1,682 (45.0%) | 1,509 (41.4%) | 6.9x10^6^ | 8.5%  (2.8x10^-5^) | 4.9%  (0.01) | 3.6%  (0.003) |
| More social | 250 (30.3%) | 1,224 (32.8%) | 954 (26.2%) | 3.6x10^-9^ | 2.5%  (0.2) | 4.1%  (0.03) | 6.6%  (1.8x10^-9^) |
| More active | 426 (51.6%) | 1,857 (49.7%) | 1,573 (43.1%) | 2.0x10^-9^ | 1.9%  (0.3) | 8.5%  (1.6x10^-5^) | 6.6%  (5.1x10^-8^) |
| More energy | 376 (45.6%) | 1,836 (49.1%) | 1,484 (40.7%) | 2.5x10-12 | 3.5%  (0.07) | 4.9%  (0.02) | 8.4% (1.0x10^-12^) |
| Concentration difficulties | 576 (69.8%) | 2,959 (79.2%) | 2,870 (78.7%) | 1.4x10^-8^ | 9.4%  (2.2x10^-8^) | 8.9%  (9.2x10^-8^) | 0.5%  (0.5) |
| Racing thoughts | 598 (72.5%) | 2,980 (79.7%) | 2,878 (78.9%) | 2.2x10^-5^ | 7.2%  (1.7x10^-5^) | 6.4%  (1.2x10^-4^) | 0.8%  (0.4) |
| More talkative | 372 (45.1%) | 2,110 (56.5%) | 1,839 (50.4%) | 3.7x10^-11^ | 11.4%  (1.1x10^-8^) | 5.3%  (0.007) | 6.1%  (3.2x10^-7^) |
| Less sleep | 395 (47.9%) | 1,985  (53.1%) | 1,623 (44.5%) | 9.9x10^-13^ | 5.2%  (0.01) | 3.4%  (0.08) | 8.6% (4.4x10^-13^) |
| Irritability | 551 (66.8%) | 2,882 (77.1%) | 2,848 (78.1%) | 2.2x10^-11^ | 10.3%  (9.6x10^-10^) | 11.3%  (3.0x10^-11^) | 1%  (0.3) |
| Hyperactivity | 331 (40.1%) | 1,838 (49.2%) | 1,543 (42.3%) | 1.7x10^-10^ | 9.1%  (4.3x10^-6^) | 2.2%  (0.3) | 6.9%  (9.9x10^-9^) |
| More self-confidence | 319 (38.7%) | 1,741 (46.6%) | 1,577 (43.2%) | 4.0x10^-5^ | 7.9%  (1.2x10^-4^) | 4.5%  (0.02) | 3.4%  (0.01) |

Note: ANBP = anorexia nervosa binge-eating/purging, BN = bulimia nervosa, BED = binge-eating disorder. P-value threshold for significance of difference ($\alpha=\frac{0.05}{13}=0.004$). P-values in pairwise significance of difference tests are FDR-adjusted, with $\alpha$ = 0.001.

**2.2.2 Edge weight and centrality difference tests**

Below are the bootstrapped edge weight difference test results and the bootstrapped difference tests of expected influence within the anorexia nervosa binge-eating/purging group, the bulimia nervosa group, and the binge-eating disorder group. Bootstrapped difference tests of non-zero edge weights indicated that, out of the maximum 105 edge weights, 50 were estimated to be non-zero in the anorexia nervosa binge-eating/purging group, 71 were estimated as non-zero in the bulimia nervosa group, and 75 in the binge-eating disorder group. Again, this probably reflects the differences in sample size.

**
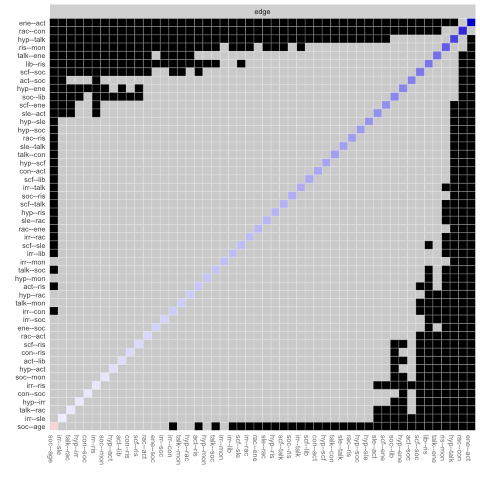
**

**SUPPLEMENTARY FIGURE 17** Bootstrapped non-zero edge weight difference tests in the anorexia nervosa binge-eating/purging group (*n* = 825). Black boxes indicate a significant difference between edge weights. Edge weights are arranged in order of strength, with the strongest edge weight on the far right of the axis. The diagonal indicates the relative strength of each edge weight, with a darker colour indicating a stronger edge weight. The colour of the diagonal corresponds to the colour of the edge weights in the network plot. As indicated by the edge weight accuracy tests, care should be taken when interpreting edge weights in the anorexia nervosa binge-eating/purging group. Blue indicates a positive association, and red indicates a negative association. Note: mon = reckless spending, irr = irritability, con = concentration difficulties, sle = less sleep, lib = higher libido, scf = more self-confidence, rac = racing thoughts, talk = more talkative, soc = more social, ris = unusual and/or risky behaviour, ene = more energy, act = more active, hyp = hyperactivity.


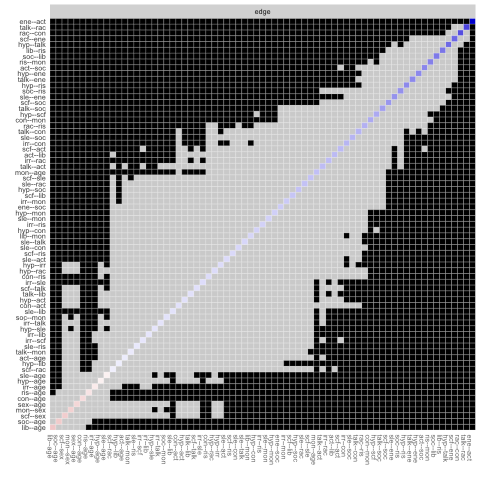


**SUPPLEMENTARY FIGURE 18** Bootstrapped non-zero edge weight difference tests in the bulimia group (*n* = 3,737). Black boxes indicate a significant difference between edge weights. Edge weights are arranged in order of strength, with the strongest edge weight on the far right of the axis. The diagonal indicates the relative strength of each edge weight, with a darker colour indicating a stronger edge weight. The colour of the diagonal corresponds to the colour of the edge weights in the network plot. Blue indicates a positive association, and red indicates a negative association. Note: mon = reckless spending, irr = irritability, con = concentration difficulties, sle = less sleep, lib = higher libido, scf = more self-confidence, rac = racing thoughts, talk = more talkative, soc = more social, ris = unusual and/or risky behaviour, ene = more energy, act = more active, hyp = hyperactivity.


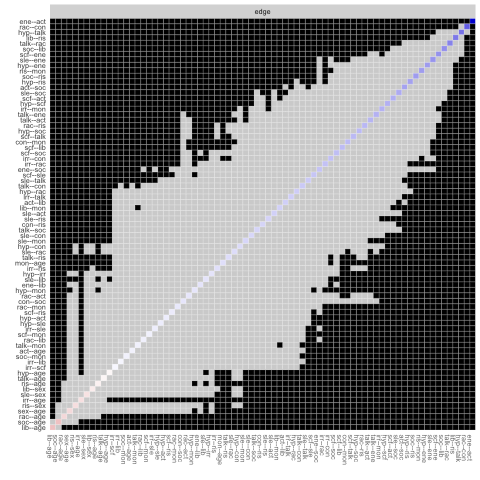


**SUPPLEMENTARY FIGURE 19** Bootstrapped non-zero edge weight difference tests in the binge-eating disorder group (*n* = 3,648). Black boxes indicate a significant difference between edge weights. Edge weights are arranged in order of strength, with the strongest edge weight on the far right of the axis. The diagonal indicates the relative strength of each edge weight, with a darker colour indicating a stronger edge weight. The colour of the diagonal corresponds to the colour of the edge weights in the network plot. Blue indicates a positive association, and red indicates a negative association. Note: mon = reckless spending, irr = irritability, con = concentration difficulties, sle = less sleep, lib = higher libido, scf = more self-confidence, rac = racing thoughts, talk = more talkative, soc = more social, ris = unusual and/or risky behaviour, ene = more energy, act = more active, hyp = hyperactivity.


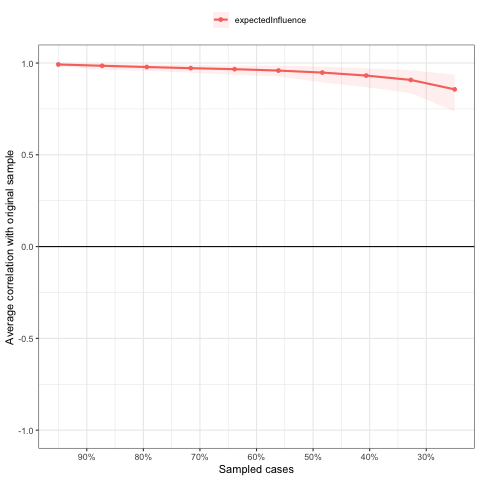


**SUPPLEMENTARY FIGURE 20** Centrality stability plot in the anorexia nervosa binge-eating/purging group (*n* = 825). Correlation between original order of expected influence with order of expected influence after case-dropping bootstrapping (i.e., dropping rows from the data).


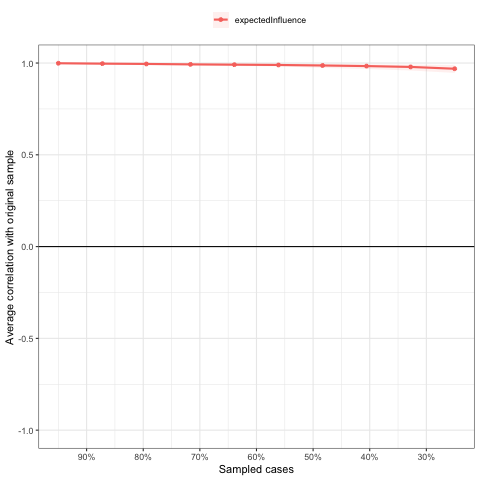


**SUPPLEMENTARY FIGURE 21** Centrality stability plot in the bulimia nervosa group (*n* = 3,737). Correlation between original order of expected influence with order of expected influence after case-dropping bootstrapping (i.e., dropping rows from the data).


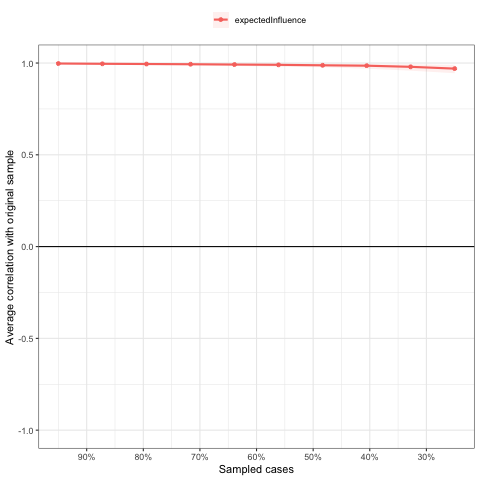


**SUPPLEMENTARY FIGURE 22** Centrality stability plot in the binge-eating disorder group (*n* = 3,648). Correlation between original order of expected influence with order of expected influence after case-dropping bootstrapping (i.e., dropping rows from the data).


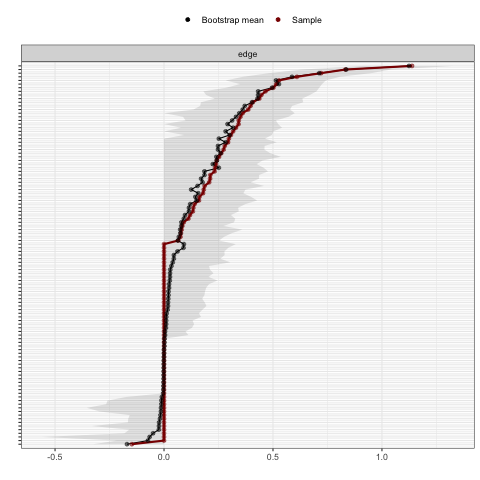


**SUPPLEMENTARY FIGURE 23 The** 95% bootstrapped confidence intervals of all non-zero edge weights in the anorexia nervosa binge-eating/purging group (*n* = 825). The Y axis represents the edge weight relationships (labels removed to avoid cluttering), with each line indicating a relationship between two edges, ordered from the lowest edge weight to the highest in ascending order. The X axis represents the weight of each relationship between two edges. Sample values are demonstrated by the red line, and the black line indicates the mean of the bootstrapped estimates.


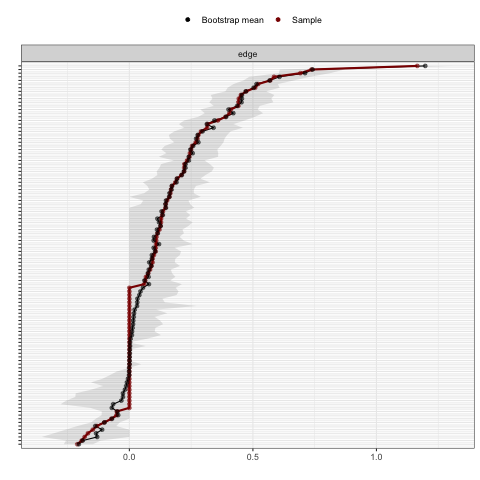


**SUPPLEMENTARY FIGURE 24 The** 95% bootstrapped confidence intervals of all non-zero edge weights in the bulimia nervosa group (*n* = 3,737). The Y axis represents the edge weight relationships (labels removed to avoid cluttering), with each line indicating a relationship between two edges, ordered from the lowest edge weight to the highest in ascending order. The X axis represents the weight of each relationship between two edges. Sample values are demonstrated by the red line, and the black line indicates the mean of the bootstrapped estimates.

**
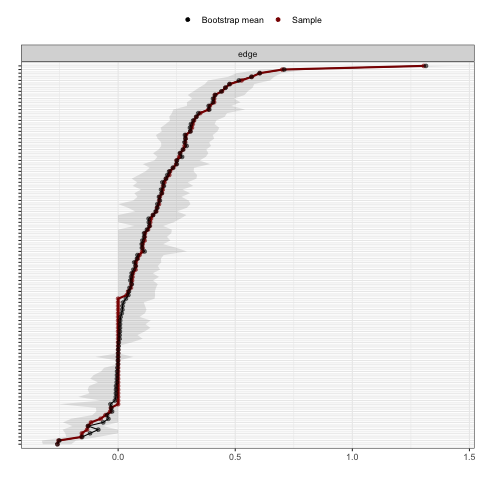
**

**SUPPLEMENTARY FIGURE 25 The** 95% bootstrapped confidence intervals of all non-zero edge weights in the binge-eating disorder group (*n* = 3,648). The Y axis represents the edge weight relationships (labels removed to avoid cluttering), with each line indicating a relationship between two edges, ordered from the lowest edge weight to the highest in ascending order. The X axis represents the weight of each relationship between two edges. Sample values are demonstrated by the red line, and the black line indicates the mean of the bootstrapped estimates.


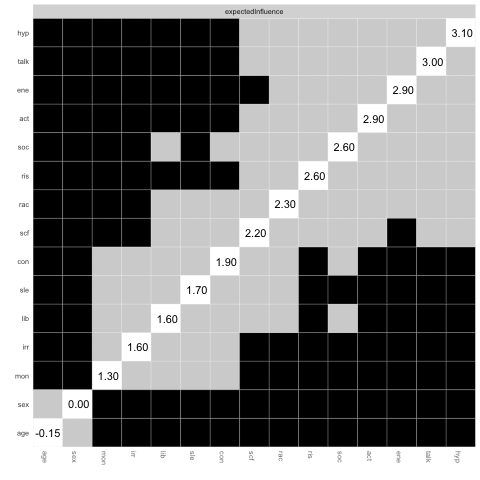


**SUPPLEMENTARY FIGURE 26** Bootstrapped difference tests of the expected influence of nodes (i.e., sum of edge weights) in the anorexia nervosa binge-eating/purging group (*n* = 825). Black boxes indicate a significant difference between nodes’ centrality estimates. Nodes are arranged in order of centrality, with the most central node on the far right of the axis. Note: mon = reckless spending, irr = irritability, con = concentration difficulties, sle = less sleep, lib = higher libido, scf = more self-confidence, rac = racing thoughts, talk = more talkative, soc = more social, ris = unusual and/or risky behaviour, ene = more energy, act = more active, hyp = hyperactivity.


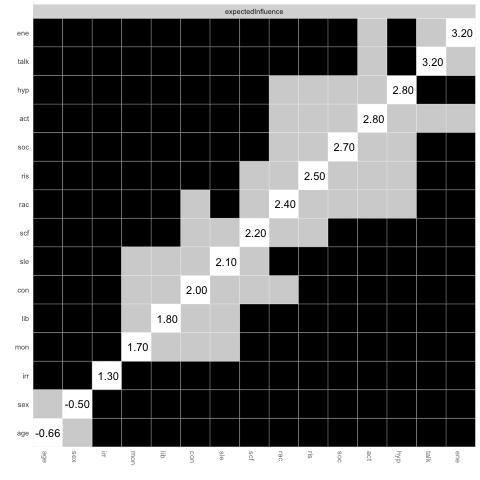


**SUPPLEMENTARY FIGURE 27** Bootstrapped difference tests of the expected influence of nodes (i.e., sum of edge weights) in the bulimia group (*n* = 3,737). Black boxes indicate a significant difference between nodes’ centrality estimates. Nodes are arranged in order of centrality, with the most central node on the far right of the axis. Note: mon = reckless spending, irr = irritability, con = concentration difficulties, sle = less sleep, lib = higher libido, scf = more self-confidence, rac = racing thoughts, talk = more talkative, soc = more social, ris = unusual and/or risky behaviour, ene = more energy, act = more active, hyp = hyperactivity.


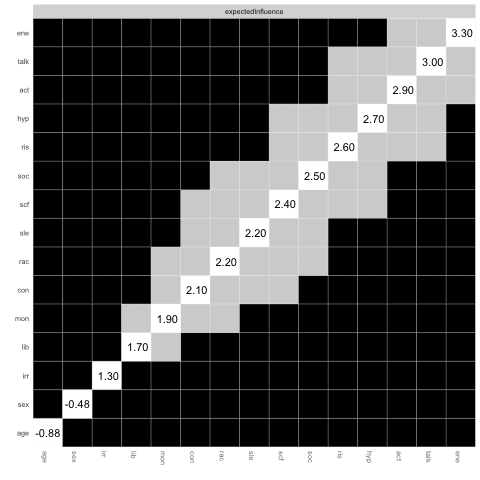


**SUPPLEMENTARY FIGURE 28** Bootstrapped difference tests of the expected influence of nodes (i.e., sum of edge weights) in the binge-eating disorder group (*n* = 3,648). Black boxes indicate a significant difference between nodes’ centrality estimates. Nodes are arranged in order of centrality, with the most central node on the far right of the axis. Note: mon = reckless spending, irr = irritability, con = concentration difficulties, sle = less sleep, lib = higher libido, scf = more self-confidence, rac = racing thoughts, talk = more talkative, soc = more social, ris = unusual and/or risky behaviour, ene = more energy, act = more active, hyp = hyperactivity.

**2.2.3 Network connectivity**

**B**ootstrapped difference tests revealed that in all three networks, the strongest edges were between 'more energy' and 'more active' (edge weights = 1.14-1.31). As stated in the main manuscript, the edge weight between ‘more talkative’ and ‘racing thoughts’ was significantly stronger in the bulimia nervosa group than in the anorexia nervosa binge-eating/purging group (*p* < 0.001).

**2.2.4 Node accuracy**

Supplementary Table 6 below shows the normalised accuracy[^9^](https://paperpile.com/c/sZAmz0/Nt6I) of each node in the networks within the diagnosis-level analysis.

**SUPPLEMENTARY TABLE 6** Normalised accuracy of each node in networks of people with binge-type eating disorders (anorexia nervosa binge-eating/purging *n* = 825; bulimia nervosa *n* = 3,737; binge-eating disorder *n* = 3,648). Participants are from the National Institute for Health and Care Research (NIHR) BioResource (*n* = 8,210). Diagnoses have been assigned using self-report (via the Mental Health Diagnosis [MHD] questionnaire) and/or diagnostic algorithms based on the Diagnostic and Statistical Manual of Mental Disorders, Fifth Edition (DSM-5; via the ED100K) in either the Genetic Links to Anxiety and Depression (GLAD) Study, the Eating Disorders Genetics Initiative (EDGI UK), and/or the COVID-19 Psychiatry and Neurological Genetics (COPING) study. Participants are a sub-sample of the people who report binge eating.

|  | **Anorexia nervosa binge-eating/purging** | **Bulimia nervosa** | **Binge-eating disorder** |
| --- | --- | --- | --- |
| Hyperactivity | 0.61 | 0.62 | 0.53 |
| Irritability | 0.24 | 0.09 | 0.08 |
| More self-confidence | 0.52 | 0.55 | 0.53 |
| Less sleep | 0.48 | 0.47 | 0.48 |
| More talkative | 0.61 | 0.58 | 0.59 |
| Racing thoughts | 0.40 | 0.28 | 0.27 |
| Concentration difficulties | 0.37 | 0.23 | 0.22 |
| More energy | 0.70 | 0.72 | 0.70 |
| More active | 0.66 | 0.70 | 0.69 |
| More social | 0.45 | 0.49 | 0.41 |
| Higher libido | 0.42 | 0.48 | 0.46 |
| Unusual and/or risky behaviour | 0.50 | 0.52 | 0.50 |
| Reckless spending | 0.04 | 0.29 | 0.32 |


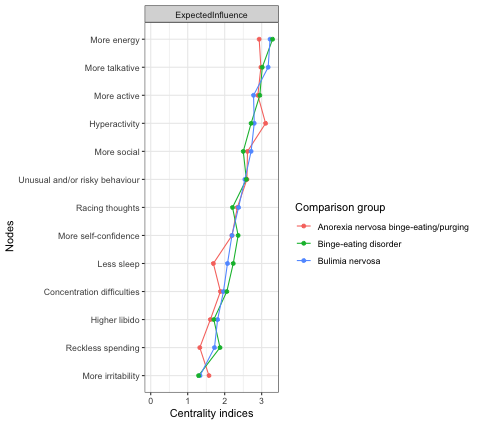


**SUPPLEMENTARY FIGURE 29** Centrality plots comparing the standardised expected influence of each symptom node in the groups in the diagnosis-level analysis (anorexia nervosa binge-eating/purging *n* = 825; bulimia nervosa *n* = 3,737; binge-eating disorder *n* = 3,648).

**3 RESULTS: SENSITIVITY ANALYSES**

**3.1 Pre-processing checks**

Across all sensitivity analyses, pre-processing checks indicated that no nodes measured the same underlying construct. No nodes consistently had near-zero-variance or zero-variance across all groups in the relevant comparisons, therefore we kept all nodes in all models as networks with different nodes cannot be compared.

**3.2 Network stability and accuracy**

In all sensitivity analyses, bootstrapped expected influence CS-coefficients showed high stability (CS-coefficients > 0.5) and plots generated through the bootstrapping procedure indicated we were able to interpret differences in centrality using expected influence[^2^](https://paperpile.com/c/sZAmz0/Su8nU). However, the confidence intervals generated via the bootstrapping tests indicated that the edge weights in networks with samples of below 1,000 participants were estimated with lower precision, likely due to their smaller sample sizes, thus care must be taken when interpreting results in such networks. Within each sensitivity analyses, we used the same *p*-value threshold as in the main analysis (α *=* 0.0125). We generated Bonferroni-adjusted p-values for the differences in the specific edge weights and centrality of nodes across networks.

**3.3 Restricted to people with at least one mania symptom**

**3.3.1 Centrality.**

In the binge eating and no binge eating network, ‘more talkative’ and ‘more energy’ were the most central and ‘irritability’ was the least central (Supplementary Figure 30). A network comparison test indicated that the symptom ‘less sleep’ was significantly more central in the binge eating group than the no binge eating group (*p* < 0.001).

In the diagnosis-level analysis, ‘irritability’ was the least central across all networks (Supplementary Figure 31). ‘More energy’ and ‘more talkative’ were the most central symptoms in the bulimia nervosa and binge-eating disorder network. In the anorexia nervosa binge-eating/purging network, the most central symptoms were ‘hyperactivity’ and ‘more active’. None of the differences in centrality between the groups in the diagnosis-level analysis were significant.


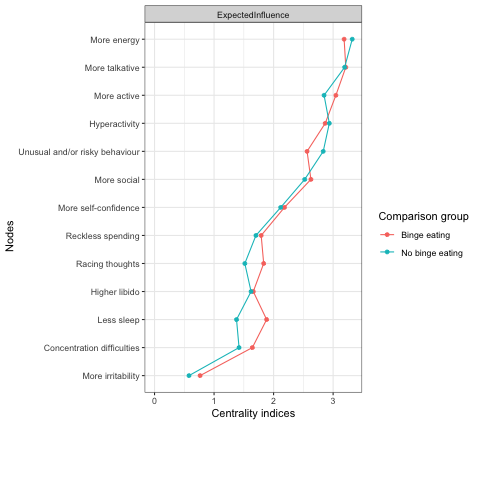


**SUPPLEMENTARY FIGURE 30** Centrality plot comparing the standardised expected influence of each symptom node in the groups within the symptom-level analysis (binge eating *n* = 11,411; no binge eating *n* = 14,696) who endorsed at least one mania symptom.

**
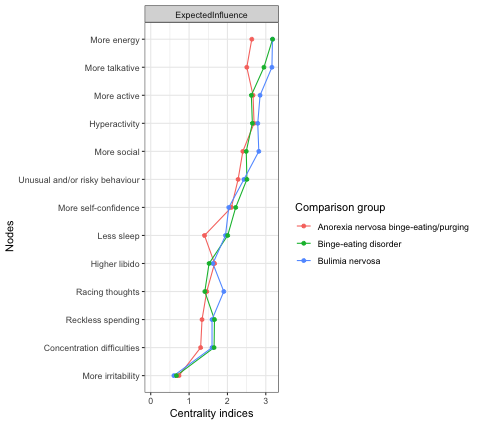
**

**SUPPLEMENTARY FIGURE 31** Centrality plot comparing the standardised expected influence of each symptom node in the groups within the diagnosis-level analysis (anorexia nervosa binge-eating/purging *n* = 751; bulimia nervosa = 3,576; binge-eating disorder *n* = 3,488) who endorsed at least one mania symptom.

**3.3.2 Node accuracy**

In the symptom-level analysis, the nodes ‘more energy’ (0.69 and 0.50) and ‘more active’ (0.68 and 0.55) had the highest normalised accuracy in the binge eating network and the no binge eating network (Supplementary Table 7). ‘Irritability’ had low normalised accuracy in the binge eating network and the no binge eating network, with values of 0 and 0.10, respectively. ‘Reckless spending’ had the lowest normalised accuracy in the no binge eating network (0.05).

In the anorexia nervosa binge-eating/purging, bulimia nervosa, and binge-eating disorder networks, the node ‘more energy’ (0.69, 0.71, and 0.69) had high normalised accuracy (Supplementary Table 8). ‘Irritability’ had low normalised accuracy in the anorexia nervosa binge-eating/purging network (0), bulimia nervosa network (0), and the binge-eating disorder network (0).

**SUPPLEMENTARY TABLE 7** Normalised accuracy of each node in the binge eating group (*n* = 11,411) and the no binge eating group (*n* = 14,696) in the sensitivity analysis in which we restricted to participants with at least one mania symptom. Participants are from the National Institute for Health and Care Research (NIHR) BioResource. The presence or absence of lifetime binge eating was assessed in either the Genetic Links to Anxiety and Depression (GLAD) Study, the Eating Disorders Genetics Initiative (EDGI UK), and/or the COVID-19 Psychiatry and Neurological Genetics (COPING) study.

|  | **Normalised accuracy** | |
| --- | --- | --- |
|  | **Binge eating** | **No binge eating** |
| Hyperactivity | 0.55 | 0.32 |
| Irritability | 0.00 | 0.10 |
| More self-confidence | 0.51 | 0.30 |
| Less sleep | 0.47 | 0.19 |
| More talkative | 0.57 | 0.35 |
| Racing thoughts | 0.09 | 0.22 |
| Concentration difficulties | 0.05 | 0.26 |
| More energy | 0.69 | 0.50 |
| More active | 0.68 | 0.55 |
| More social | 0.43 | 0.22 |
| Higher libido | 0.43 | 0.22 |
| Unusual and/or risky behaviour | 0.50 | 0.25 |
| Reckless spending | 0.22 | 0.05 |

**SUPPLEMENTARY TABLE 8** Normalised accuracy of each node in networks of people with binge-type eating disorders (anorexia nervosa binge-eating/purging *n* = 751; bulimia nervosa = 3,576; binge-eating disorder *n* = 3,488) in the sensitivity analysis in which we restricted to participants with at least one mania symptom. Participants are from the National Institute for Health and Care Research (NIHR) BioResource. Diagnoses have been assigned using self-report (via the Mental Health Diagnosis [MHD] questionnaire) and/or diagnostic algorithms based on the Diagnostic and Statistical Manual of Mental Disorders, Fifth Edition (DSM-5; via the ED100K) in either the Genetic Links to Anxiety and Depression (GLAD) Study, the Eating Disorders Genetics Initiative (EDGI UK), and/or the COVID-19 Psychiatry and Neurological Genetics (COPING) study. Participants are a sub-sample of the people who report binge eating.

|  | **Anorexia nervosa binge-eating/purging** | **Bulimia nervosa** | **Binge-eating disorder** |
| --- | --- | --- | --- |
| Hyperactivity | 0.60 | 0.59 | 0.53 |
| Irritability | 0.00 | 0.00 | 0.00 |
| More self-confidence | 0.51 | 0.55 | 0.53 |
| Less sleep | 0.43 | 0.42 | 0.48 |
| More talkative | 0.61 | 0.53 | 0.55 |
| Racing thoughts | 0.09 | 0.07 | 0.06 |
| Concentration difficulties | 0.10 | 0.05 | 0.01 |
| More energy | 0.69 | 0.71 | 0.69 |
| More active | 0.59 | 0.68 | 0.70 |
| More social | 0.45 | 0.49 | 0.41 |
| Higher libido | 0.42 | 0.47 | 0.45 |
| Unusual and/or risky behaviour | 0.51 | 0.48 | 0.50 |
| Reckless spending | 0.06 | 0.29 | 0.30 |

**3.3.3 Network comparison**

Networks of the symptom groups (no binge eating *n* = 14,696; binge eating *n* = 11,411) revealed no significant differences in global strength (S = 0.34, *p* = 0.48) but a significant difference in network structure (M = 0.22, *p* < 0.001; Supplementary Figure 32). The edge weights between: ‘irritability’ with ‘less sleep’, ‘irritability’ with ‘racing thoughts’, ‘concentration difficulties’ with ‘less sleep’, and ‘concentration difficulties’ with ‘more active’ were significantly stronger in the binge eating network (all *p*s < 0.001), whilst the edge weight between ‘more self-confidence’ and ‘more talkative’ was significantly stronger in the no binge eating network (*p* < 0.001).

Networks of the diagnosis groups (anorexia nervosa binge-eating/purging *n* = 751; bulimia nervosa = 3,576; binge-eating disorder *n* = 3,488) revealed no significant differences in global strength (S = 0.89-3.46, all *p*s > 0.1) across all networks (Supplementary Figure 33). Whilst we found no significant differences in network structure when comparing binge-eating disorder with bulimia nervosa (M = 0.24 *p* = 0.23), we found the network structure of the anorexia nervosa binge-eating/purging group was significantly different to the binge-eating disorder (M = 0.49, *p* = 0.008) and bulimia nervosa groups (M = 0.73, *p* < 0.001). Regarding this former finding, however, the edge weight invariance test indicated that this significant difference in network structure was not the result of the same single or multiple edge weight/s each time, i.e., the result was not consistent and was a product of spurious differences across different edges. Regarding the latter finding, the edge weight between ‘more talkative’ and ‘racing thoughts’ was consistently and significantly stronger in the bulimia nervosa network (0.73) than in the anorexia nervosa binge-eating/purging network (0, *p* < 0.001).

**
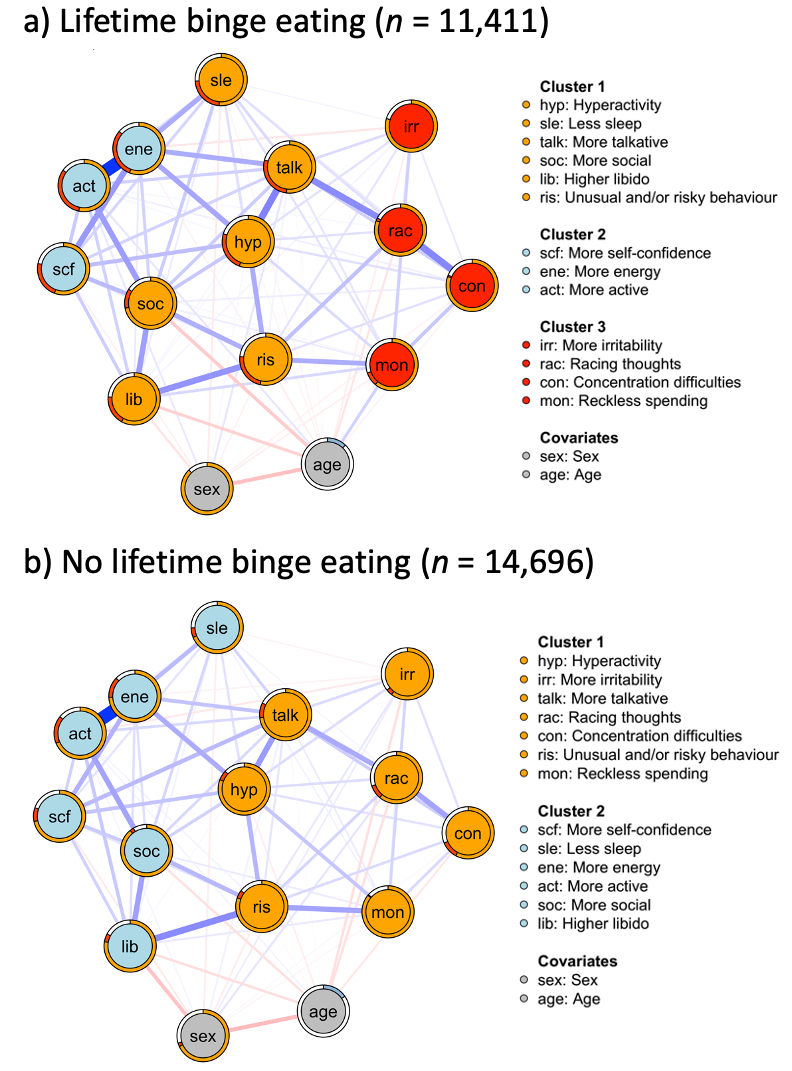
**

**SUPPLEMENTARY FIGURE 32** Mania symptom networks in individuals with (32a; *n* = 11,411) and without (32b; *n* = 14,696) lifetime binge eating, who endorsed at least one mania symptom. Blue edges indicate positive associations and red edges indicate negative associations. The width and saturation of the edge indicates strength of the relationship, with thicker and more saturated edges representing stronger associations. Networks are plotted by calculating the average layout of the networks, and then constraining each of these networks to that layout. Within each network, the colour of the node indicates its cluster membership as defined by the walktrap algorithm (covariates have been forced into their own category). For all binary nodes, the orange colour around each node indicates the accuracy achieved by the marginal (i.e., unadjusted model); the red colour around each node indicates the additional accuracy achieved by all nodes that are connected to that node. Red + orange denotes the accuracy of the full model (i.e., marginal + additional accuracy). Normalised accuracy is depicted by the ratio of red/(red + white). Normalised accuracy is the accuracy achieved by all nodes it is connected to, beyond the accuracy achieved by the marginal. For the continuous node (i.e., age), the blue bar indicates the explained variance achieved by all nodes it is connected to.


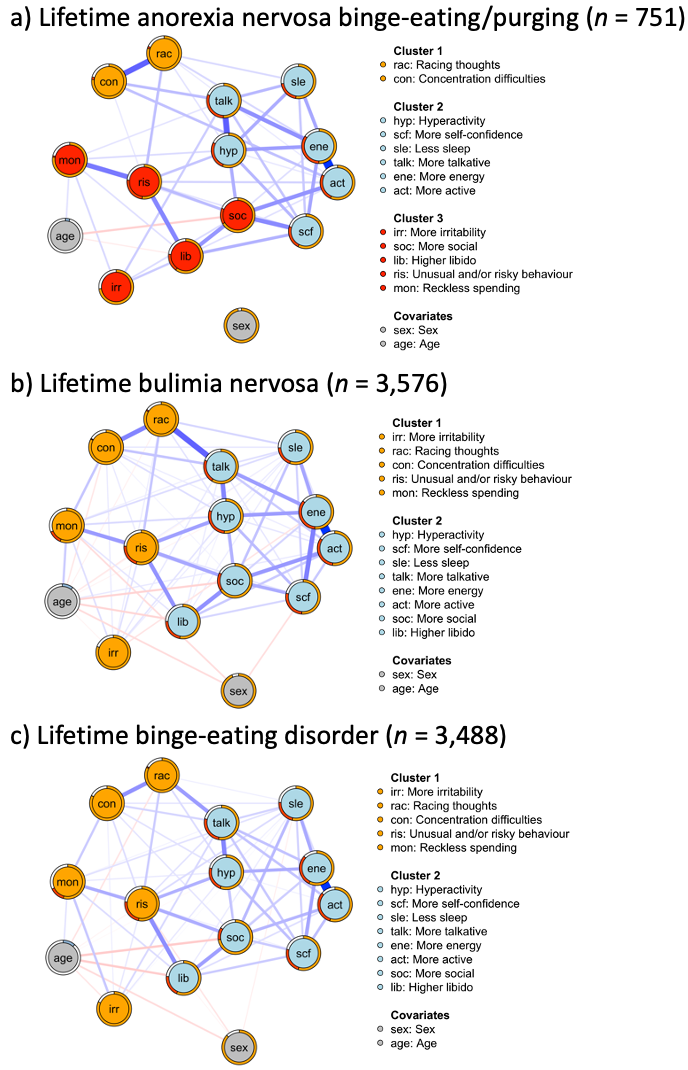


**SUPPLEMENTARY FIGURE 33** Mania symptom networks in individuals hierarchically categorised into groups of lifetime diagnosis of anorexia nervosa binge-eating/purging (33a; *n* = 751), bulimia nervosa (33b; *n* = 3,576), binge-eating disorder (34c; *n* = 3,488), who endorsed at least one mania symptom. Blue edges indicate positive associations and red edges indicate negative associations. The width and saturation of the edge indicates strength of the relationship, with thicker and more saturated edges representing stronger associations. Networks are plotted by calculating the average layout of the networks, and then constraining each of these networks to that layout. Within each network, the colour of the node indicates its cluster membership as defined by the walktrap algorithm (covariates have been forced into their own category). For all binary nodes, the orange colour around each node indicates the accuracy achieved by the marginal (i.e., unadjusted model); the red colour around each node indicates the additional accuracy achieved by all nodes that are connected to that node. Red + orange denotes the accuracy of the full model (i.e., marginal + additional accuracy). Normalised accuracy is depicted by the ratio of red/(red + white). Normalised accuracy is the accuracy achieved by all nodes it is connected to, beyond the accuracy achieved by the marginal. For the continuous node (i.e., age), the blue bar indicates the explained variance by all nodes it is connected to.

**3.4 Symptom-level analysis: Down-sampling to the size of the anorexia nervosa binge-eating/purging group (*n* = 825) and the binge-eating disorder group (*n* = 3,648)**

In our down-sampling of the binge eating and no binge eating group to the size of the smallest group (*n* = 825) and the second-smallest group (*n* = 3,648) in the diagnosis-level analysis, we aimed to assess whether we still found significant differences in global strength and/or network structure. Therefore, for these analyses, we present only the network comparison test results.

**3.4.1 Network comparison test**

Networks of the symptom groups down-sampled to the smallest group in the diagnosis group (i.e., to the size of the ANBP group, *n* = 825) revealed significant differences in global strength (binge eating = 12.79; no binge eating = 16.57; *p* < 0.001) but not in network structure (M = 0.67, *p* = 0.03) (Supplementary Figure 34). Similarly, when we down-sampled to the second smallest group in the diagnosis group (i.e., to the size of the BED group, *n* = 3,648; Supplementary Figure 35), we found significant differences in global strength (binge eating = 17.21; no binge eating = 19.71, *p* < 0.001) but not in network structure (M = 0.34, *p* = 0.03).

**
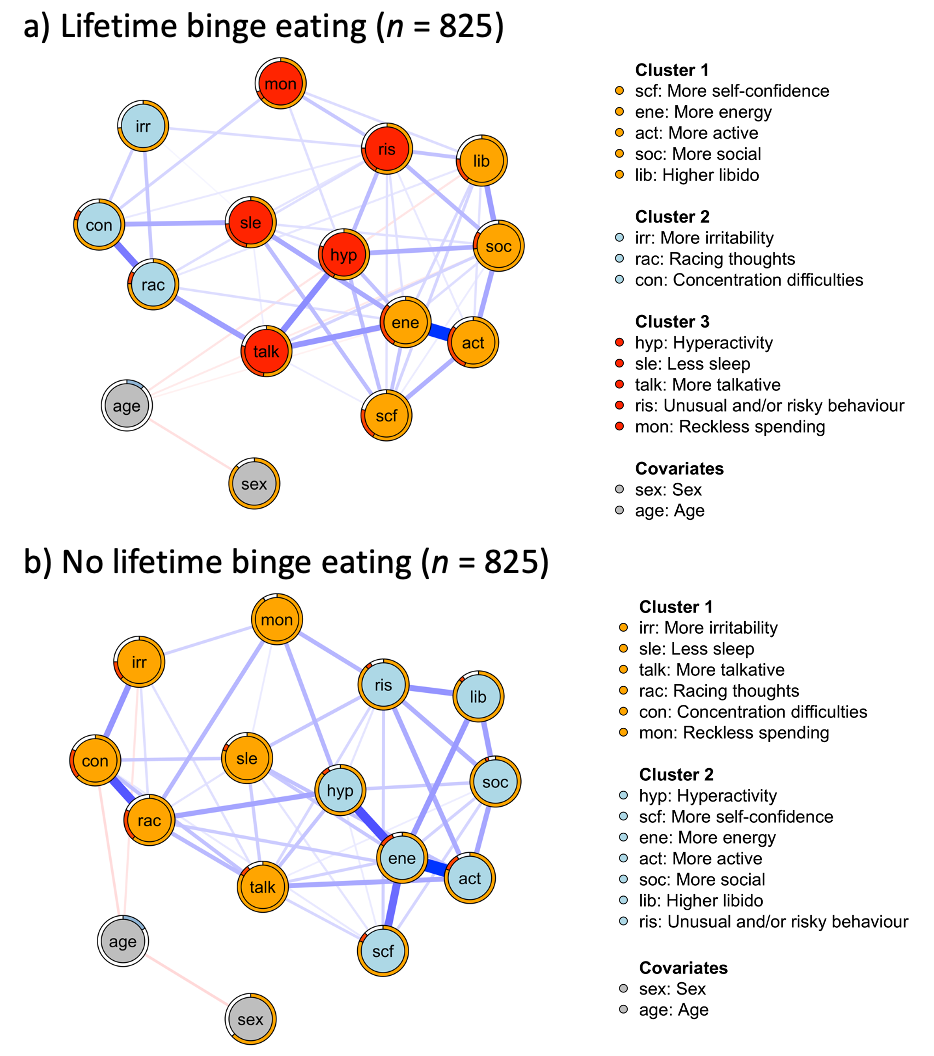
**

**SUPPLEMENTARY FIGURE 34** Mania symptom networks in individuals with (34a; *n* = 825) and without (34b; *n* = 825) lifetime binge eating, down-sampled to the size of the smallest group in the diagnosis-level analysis. Blue edges indicate positive associations and red edges indicate negative associations. The width and saturation of the edge indicates strength of the relationship, with thicker and more saturated edges representing stronger associations. Networks are plotted by calculating the average layout of the networks, and then constraining each of these networks to that layout. Within each network, the colour of the node indicates its cluster membership as defined by the walktrap algorithm (covariates have been forced into their own category). For all binary nodes, the orange colour around each node indicates the accuracy achieved by the marginal (i.e., unadjusted model); the red colour around each node indicates the additional accuracy achieved by all nodes that are connected to that node. Red + orange denotes the accuracy of the full model (i.e., marginal + additional accuracy). Normalised accuracy is depicted by the ratio of red/(red + white). Normalised accuracy is the accuracy achieved by all nodes it is connected to, beyond the accuracy achieved by the marginal. For the continuous node (i.e., age), the blue bar indicates the explained variance achieved by all nodes it is connected to.

**
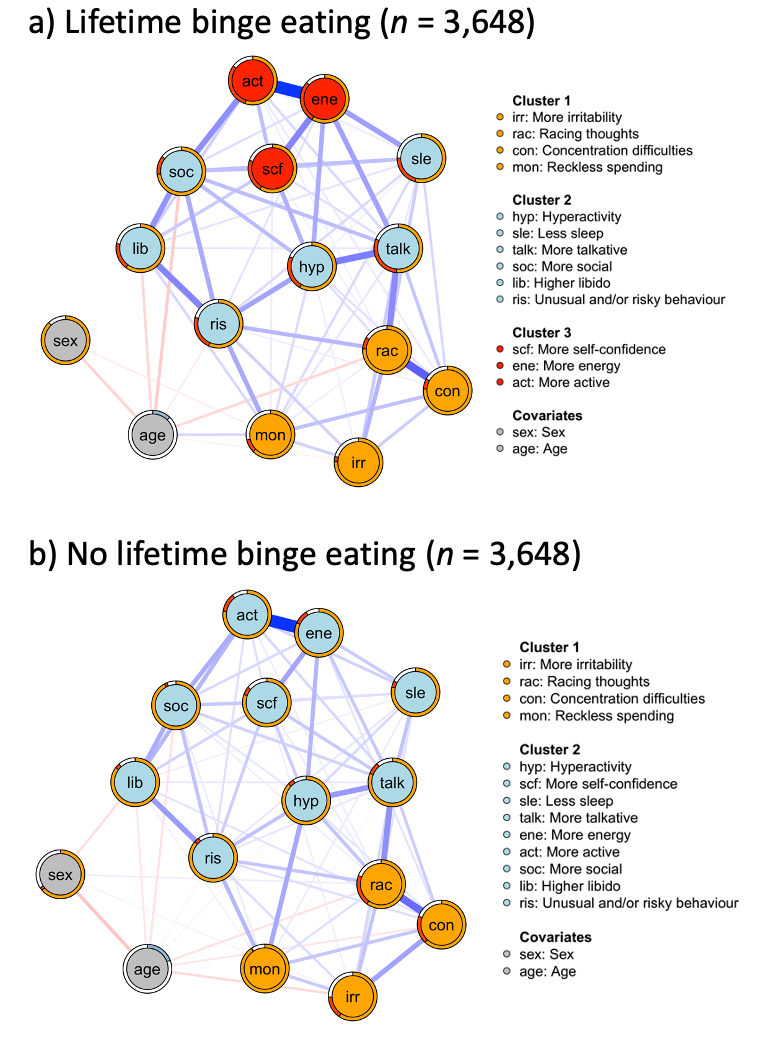
**

**SUPPLEMENTARY FIGURE 35** Mania symptom networks in individuals with (35a; *n* = 3,648) and without (35b; *n* = 3,648) lifetime binge eating, down-sampled to the size of the second-smallest group in the diagnosis-level analysis. Blue edges indicate positive associations and red edges indicate negative associations. The width and saturation of the edge indicates strength of the relationship, with thicker and more saturated edges representing stronger associations. Networks are plotted by calculating the average layout of the networks, and then constraining each of these networks to that layout. Within each network, the colour of the node indicates its cluster membership as defined by the walktrap algorithm (covariates have been forced into their own category). For all binary nodes, the orange colour around each node indicates the accuracy achieved by the marginal (i.e., unadjusted model); the red colour around each node indicates the additional accuracy achieved by all nodes that are connected to that node. Red + orange denotes the accuracy of the full model (i.e., marginal + additional accuracy). Normalised accuracy is depicted by the ratio of red/(red + white). Normalised accuracy is the accuracy achieved by all nodes it is connected to, beyond the accuracy achieved by the marginal. For the continuous node (i.e., age), the blue bar indicates the explained variance achieved by all nodes it is connected to.

**3.5 Symptom-level analysis: Overeating (i.e., no loss of control)**

In the below sensitivity analysis, we have included participants who were not in any of the analyses in the main manuscript (926 people with overeating). Therefore, we have included a sample description.

**3.5.1 Sample description**

The overeating group were significantly younger (median = 41 years) than the no binge eating/overeating group (median = 55 years), and significantly older than the binge eating group (median = 36 years; Supplementary Table 9). The overeating group had a significantly lower proportion of female participants (63.6%) than the binge eating group (87.8%). The groups also differed significantly on the proportion of participants who endorsed many mania symptoms (Supplementary Table 9; Figure 6).

**SUPPLEMENTARY TABLE 9** Characteristics of the no lifetime binge eating (*n* = 22,122), binge eating (*n* = 12,104), and lifetime overeating (*n* = 926) from the National Institute for Health and Care Research (NIHR) BioResource. Participants indicated the presence or absence of lifetime binge eating with loss of control in either the Genetic Links to Anxiety and Depression (GLAD) Study, the Eating Disorders Genetics Initiative (EDGI UK), and/or the COVID-19 Psychiatry and Neurological Genetics (COPING) study.

|  |  | |  |  |  | **Difference**  **(Pairwise significance of difference)** | | |
| --- | --- | --- | --- | --- | --- | --- | --- | --- |
|  | **No BE or overeating** | | **BE**  **(i.e., with loss of control)** | **Overeating (i.e., no loss of control)** | **Significance of difference test** | **No BE or overeating vs. BE** | **No BE or overeating vs. Overeating** | **BE vs. Overeating** |
| Total | 22,122 | | 12,104 | 926 |  |  |  |  |
| Age (median, IQR) | 55  (23) | | 36  (23) | 41  (23) | 2.1x10^-16^ | 19  (2.1x10^-16^) | 14  (2.2x10^-74^) | 5  (5.3x10^-14^) |
| Being female | 14,166  (64.0%) | | 10,625  (87.8%) | 589  (63.6%) | 2.6x10^-16^ | 23.8%  (2.1x10^-16^) | 0.4%  (0.82) | 24.2%  (1.6x10^-92^) |
| AS levels or higher | 16,221 (74.6%)† | | 9,370  (78.1%)† | 670  (73.2%)† | 4.6x10^-35^ | 3.5%  (1.8x10^-12^) | 1.4%  (0.37) | 4.9%  (0.001) |
| Racially minoritised | 577  (2.7%)† | | 572  (4.8%)† | 32  (3.5%)† | 1.2x10^-22^ | 2.1%  (3.7x10^-23^) | 0.8%  (0.15) | 1.3%  (0.15) |
| Lowest lifetime BMI [kg/m2] (median, IQR) | 23.6  (5.7) | | 24.2  (7.7) | 24.2  (6.7) | 2.6x10^-16^ | 0.6  (1.7x10^-15^) | 0.6  (0.001) | 0  (0.60) |
| Highest lifetime BMI [kg/m2]  (median, IQR) | 30.0  (7.9) | | 34.9  (13.0) | 32.8  (10.9) | 2.6x10^-16^ | 4.9 (2.6x10^-16^) | 2.8 (3.8x10^-26^) | 2.1 (1.0x10^-7^) |
| BMI at registration [kg/m2]  (median, IQR) | 28.2  (7.4) | | 31.6  (12.3) | 30.1  (9.4) | 4.4x10^-319^ | 3.4 (2.1x10^-16^) | 1.9 (2.1x10^-17^) | 1.5 (2.0x10^-5^) |
| Reckless spending | 2,078  (9.4%) | | 4,428  (36.6%) | 235  (25.4%) | 2.1x10^-16^ | 27.2%  (2.1x10^-16^) | 16.0%  (4.4x10^-56^) | 11.2%  (9.1x10^-12^) |
| Unusual and/or behaviour | 2,901  (13.1%) | | 5,304  (43.8%) | 303  (32.4%) | 2.1x10^-16^ | 30.7%  (2.1x10^-16^) | 19.6%  (1.6x10^-63^) | 11.1%  (6.2x10^-11^) |
| Higher libido | 3,272  (14.8%) | | 4,797  (39.6%) | 300  (32.4%) | 2.1x10^-16^ | 24.8%  (2.1x10^-16^) | 17.6%  (3.3x10^-47^) | 7.2%  (1.6x10^-5^) |
| More social | 1,601  (7.2%) | | 3,200  (26.4%) | 162  (17.5%) | 2.1x10^-16^ | 19.2%  (2.1x10^-16^) | 10.3%  (3.8x10^-30^) | 8.9%  (2.6x10^-9^) |
| More active | 4,609  (20.8%) | | 5,324  (44.0%) | 359  (38.8%) | 2.1x10^-16^ | 23.2%  (2.1x10^-16^) | 17.9%  (3x10^-38^) | 5.2%  (0.003) |
| More energy | 3,786  (17.1%) | | 5,033  (41.6%) | 336  (36.3%) | 2.1x10^-16^ | 24.5%  (2.1x10^-16^) | 19.2%  (7.7x10^-50^) | 5.3%  (0.002) |
| Concentration difficulties | 8,503  (38.4%) | | 9,185  (75.9%) | 596  (64.4%) | 2.1x10^-16^ | 37.4%  (2.1x10^-16^) | 25.9%  (6.6x10^-56^) | 11.5%  (7.8x10^-15^) |
| Racing thoughts | 8,969  (40.5%) | | 9,216  (76.1%) | 607  (65.6%) | 2.1x10^-16^ | 35.6%  (2.1x10^-16^) | 25.0%  (2.6x10^-51^) | 10.6%  (7.5x10^-13^) |
| More talkative | 3,949  (17.9%) | | 5,941  (49.1%) | 363  (39.2%) | 2.1x10^-16^ | 31.2%  (2.1x10^-16^) | 21.3%  (2.1x10^-59^) | 9.9%  (8.1x10^-9^) |
| Less sleep | | 4,589  (20.7%) | 5,554  (45.9%) | 391  (42.2%) | 2.1x10^-16^ | 25.1%  (2.1x10^-16^) | 21.5%  (3.9x10^-54^) | 3.7%  (0.03) |
| Irritability | 8,887  (40.2%) | | 9,097  (75.2%) | 621  (67.1%) | 2.1x10^-16^ | 35.0%  (2.1x10^-16^) | 26.9%  (3.3x10^-59^) | 8.1%  (6.2x10^-8^) |
| Hyperactivity | 2,823  (12.8%) | | 5,069  (41.9%) | 289  (31.2%) | 2.1x10^-16^ | 29.1%  (2.1x10^-16^) | 18.4%  (9.4x10^-58^) | 10.7%  (2.5x10^-10^) |
| More self-confidence | 4,158  (18.8%) | | 5,062  (41.8%) | 341  (36.8%) | 2.1x10^-16^ | 23.0%  (2.1x10^-16^) | 18.0%  (1.8x10^-41^) | 5.0%  (0.003) |

Note. BE = binge eating, BMI = body mass index, IQR = interquartile range. ‘Racially minoritised’ includes: Arab, Asian or Asian British, Black or Black British, and Mixed or multiple ethnic origins.

† Percentages are based on complete data, therefore may not reflect the numbers in the table. P-value threshold for significance of difference ($\alpha=\frac{0.05}{20}=0.002$). P-values in pairwise significance of difference tests are FDR-adjusted, with $\alpha$ = 0.001.

**3.5.2 Centrality**

Similar to the binge eating and no binge eating/overeating network, the nodes ‘more talkative’ and ‘more energy’ were highly central in the overeating network. The nodes ‘reckless spending’ and ‘higher libido’ were the least central in the overeating network (Supplementary Figure 36). Differences in the centrality of nodes in the overeating network versus the nodes in both the no binge eating/overeating network and the binge eating network were not statistically significant.

**
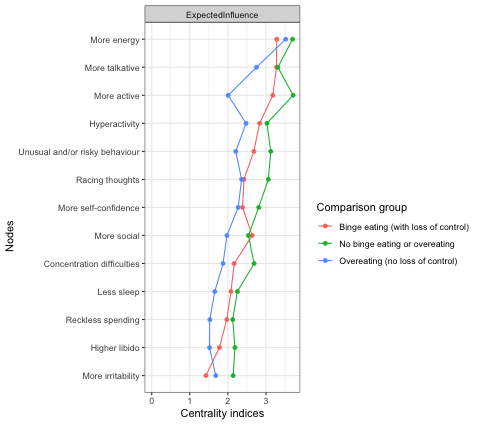
**

**SUPPLEMENTARY FIGURE 36** Centrality plot comparing the standardised expected influence of each symptom node in the groups within the symptom-level analysis, in which we have included an additional group (overeating *n* = 926; binge eating *n* = 12,104; no binge eating or overeating *n* = 22,122).

**3.5.3 Node accuracy**

Consistent with the networks in the main symptom-level analysis, ‘more energy’ (0.66) and ‘more active’ (0.66) had the highest normalised accuracy in the overeating network (Supplementary Table 10). The node ‘reckless spending’ had the lowest normalised accuracy (0.16).

**SUPPLEMENTARY TABLE 10** Normalised accuracy of each node in the no binge eating or overeating group (*n* = 22,122), the binge eating group (*n* = 12,104), and the overeating group (*n* = 926). Participants are from the National Institute for Health and Care Research (NIHR) BioResource. The presence or absence of lifetime binge eating and loss of control was assessed in either the Genetic Links to Anxiety and Depression (GLAD) Study, the Eating Disorders Genetics Initiative (EDGI UK), and/or the COVID-19 Psychiatry and Neurological Genetics (COPING) study.

|  | **Normalised accuracy** | | |
| --- | --- | --- | --- |
|  | **No binge eating or overeating** | **Binge eating (i.e., with loss of control)** | **Overeating (i.e., with no loss of control)** |
| Hyperactivity | 0.31 | 0.55 | 0.36 |
| Irritability | 0.35 | 0.13 | 0.27 |
| More self-confidence | 0.30 | 0.51 | 0.49 |
| Less sleep | 0.18 | 0.46 | 0.42 |
| More talkative | 0.35 | 0.60 | 0.48 |
| Racing thoughts | 0.50 | 0.31 | 0.43 |
| Concentration difficulties | 0.46 | 0.27 | 0.38 |
| More energy | 0.51 | 0.69 | 0.66 |
| More active | 0.55 | 0.68 | 0.66 |
| More social | 0.22 | 0.43 | 0.22 |
| Higher libido | 0.22 | 0.43 | 0.37 |
| Unusual and/or risky behaviour | 0.28 | 0.50 | 0.30 |
| Reckless spending | 0.37 | 0.23 | 0.16 |

**3.5.4 Network comparison**

Comparing the no binge eating/overeating network and the binge eating network to that of the overeating network (*n* = 926) revealed no significant differences in global strength (S = 5.75 and 3.91, *p*s > 0.1), or in network structure (M = 0.35 and 0.28, *p*s > 0.7) (Supplementary Figure 37).

**
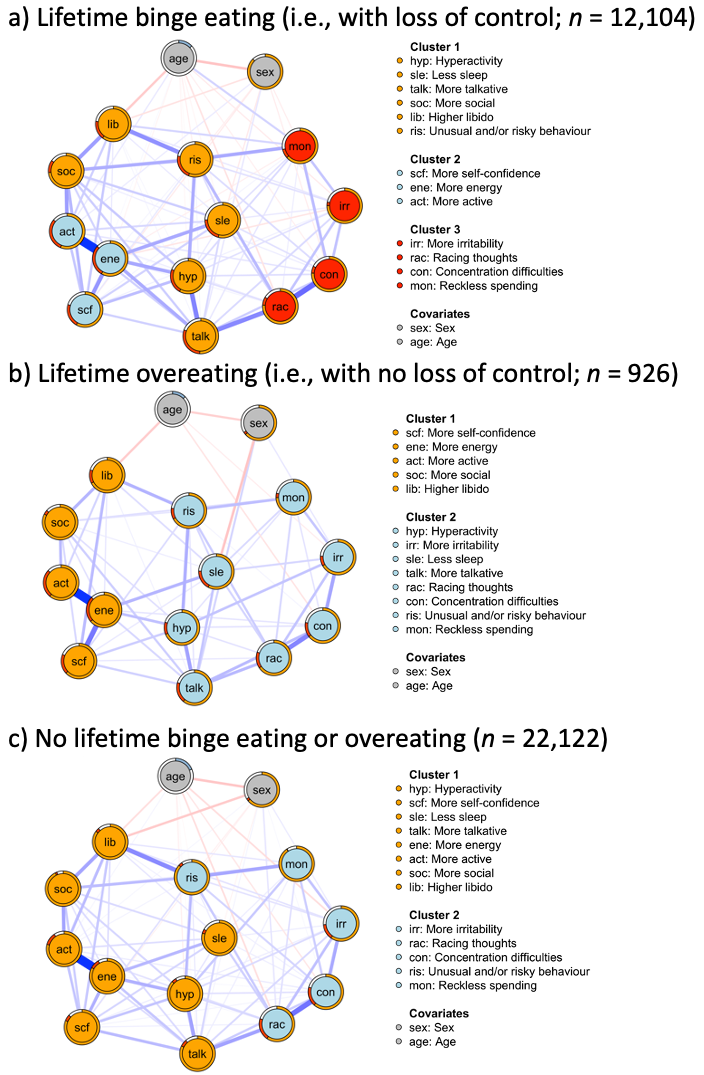
**

**SUPPLEMENTARY FIGURE 37** Mania symptom networks in individuals with lifetime binge eating (37a; *n* = 12,104), with lifetime overeating (37b; *n* = 926), and with no lifetime binge eating or overeating (37b; *n* = 22,122). Blue edges indicate positive associations and red edges indicate negative associations. The width and saturation of the edge indicates strength of the relationship, with thicker and more saturated edges representing stronger associations. Networks are plotted by calculating the average layout of the networks, and then constraining each of these networks to that layout. Within each network, the colour of the node indicates its cluster membership as defined by the walktrap algorithm (covariates have been forced into their own category). For all binary nodes, the orange colour around each node indicates the accuracy achieved by the marginal (i.e., unadjusted model); the red colour around each node indicates the additional accuracy achieved by all nodes that are connected to that node. Red + orange denotes the accuracy of the full model (i.e., marginal + additional accuracy). Normalised accuracy is depicted by the ratio of red/(red + white). Normalised accuracy is the accuracy achieved by all nodes it is connected to, beyond the accuracy achieved by the marginal. For the continuous node (i.e., age), the blue bar indicates the explained variance achieved by all nodes it is connected to.

**3.6 Diagnosis-level analysis: Single eating disorder diagnosis versus mixed presentation**

**3.6.1 Centrality**

‘Irritability’ was the least central node in the bulimia nervosa only, binge-eating disorder only and mixed presentation networks (Supplementary Figure 38). ‘More energy’ was the most central node in the mixed presentation and binge-eating disorder only networks whilst ‘more talkative’ was the most central in the bulimia nervosa only network. ‘Hyperactivity’ was the most central and ‘reckless spending’ the least central node in the anorexia nervosa binge-eating/purging only network. The differences in centrality between the nodes in the anorexia nervosa binge-eating purging network, bulimia nervosa network, binge-eating disorder network, and mixed presentation network were not statistically significant.

**
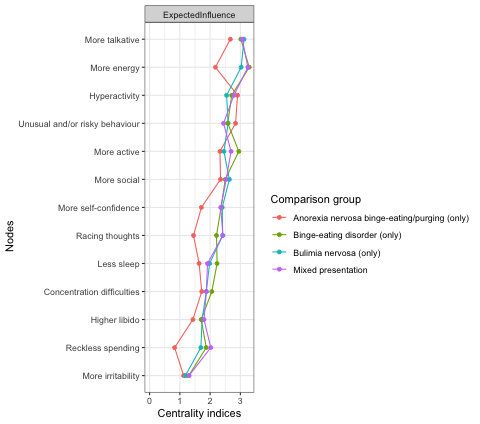
**

**SUPPLEMENTARY FIGURE 38** Centrality plot comparing the standardised expected influence of each symptom node in the groups with a single diagnosis or a mixed presentation in the diagnosis-level analysis (anorexia nervosa binge-eating/purging only *n* = 445; bulimia nervosa only *n* = 1,951; binge-eating disorder only *n* = 3,648; mixed presentation *n* = 2,166).

**3.6.2 Node accuracy**

The nodes ‘more energy’, ‘more active’, ‘hyperactivity’ and ‘more talkative’ had high normalised accuracy (0.53-0.72) across networks of people with a single eating disorder and with a mixed presentation (Supplementary Table 11). The node ‘irritability’ had the lowest normalised accuracy across all networks (0.06-0.15) other than the anorexia nervosa binge-eating/purging only network, in which ‘reckless spending’ had the lowest normalised accuracy (0).

**SUPPLEMENTARY TABLE 11** Normalised accuracy of each node in networks from our sensitivity analysis in which we grouped participants with a single diagnosis of a binge-type eating disorder (anorexia nervosa binge-eating/purging only *n* = 445; bulimia nervosa only *n* = 1,951; binge-eating disorder only *n* = 3,648) and those with more than one diagnosis of a binge-type eating disorder (mixed presentation *n* = 2,166). Participants are from the National Institute for Health and Care Research (NIHR) BioResource. Diagnoses have been assigned using self-report (via the Mental Health Diagnosis [MHD] questionnaire) and/or diagnostic algorithms based on the Diagnostic and Statistical Manual of Mental Disorders, Fifth Edition (DSM-5; via the ED100K) in either the Genetic Links to Anxiety and Depression (GLAD) Study, the Eating Disorders Genetics Initiative (EDGI UK), and/or the COVID-19 Psychiatry and Neurological Genetics (COPING) study. Participants are a sub-sample of the people who report binge eating.

|  | **Normalised accuracy** | | | |
| --- | --- | --- | --- | --- |
|  | **Anorexia nervosa binge-eating/purging** | **Bulimia nervosa** | **Binge-eating disorder** | **Mixed presentation** |
| Hyperactivity | 0.61 | 0.59 | 0.53 | 0.63 |
| Irritability | 0.29 | 0.15 | 0.08 | 0.06 |
| More self-confidence | 0.44 | 0.55 | 0.53 | 0.56 |
| Less sleep | 0.43 | 0.48 | 0.48 | 0.47 |
| More talkative | 0.62 | 0.63 | 0.59 | 0.54 |
| Racing thoughts | 0.37 | 0.31 | 0.27 | 0.26 |
| Concentration difficulties | 0.42 | 0.26 | 0.22 | 0.22 |
| More energy | 0.62 | 0.72 | 0.70 | 0.72 |
| More active | 0.62 | 0.69 | 0.69 | 0.69 |
| More social | 0.38 | 0.53 | 0.41 | 0.49 |
| Higher libido | 0.31 | 0.49 | 0.46 | 0.47 |
| Unusual and/or risky behaviour | 0.52 | 0.56 | 0.50 | 0.50 |
| Reckless spending | 0 | 0.25 | 0.32 | 0.28 |

**3.6.3 Network comparison**

Comparing the symptom networks of those with only anorexia nervosa binge-eating/purging only (*n* = 445), binge-eating disorder only (*n* = 3,648), and bulimia nervosa only (*n* = 1,951) to each other indicated no significant differences in global strength (S = 1.43-4.93, all *p*s > 0.2) or network structure (M = 0.19-0.66, all *p*s > 0.02) (Supplementary Figure 39). Comparing each of the single eating disorder presentation networks to the network of people with mixed presentation (*n* = 2,166) revealed no significant differences in global strength (S = 0.54-4.04, all *p*s > 0.3). We also found no significant differences in the network structure of the mixed presentation network compared with the bulimia nervosa only network (S = 0.23, *p* = 0.84) or with the binge-eating disorder only network (M = 0.23, *p* = 0.59). However, we found a significant difference in the network structure of the mixed presentation network compared to the anorexia nervosa binge-eating/purging only network (M = 0.68; *p* = 0.005). The edge weight between ‘more talkative’ and ‘racing thoughts’ was significantly stronger in the mixed presentation network.

**
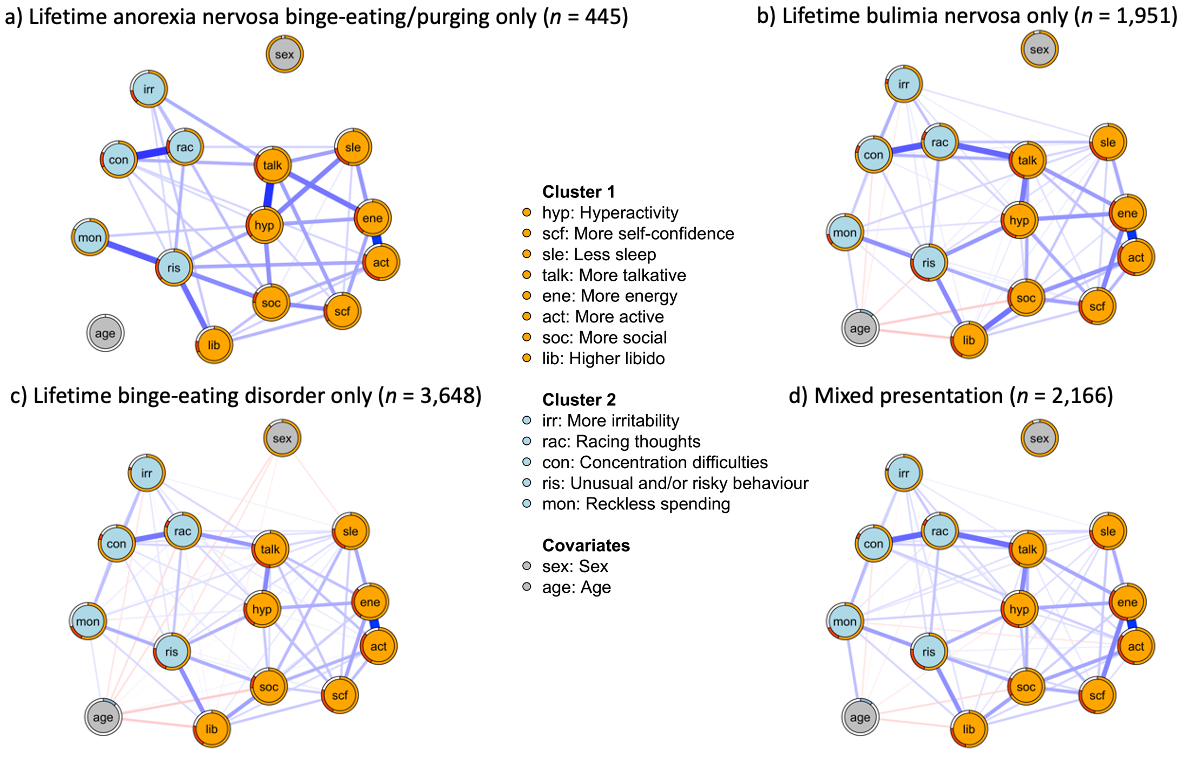
**

**SUPPLEMENTARY FIGURE 39** Mania symptom networks in individuals with a lifetime single diagnosis of anorexia nervosa binge-eating/purging only (39a; *n* = 445), bulimia nervosa only (39b; *n* = 1,951), binge-eating disorder only (39c; *n* = 3,648), and mixed presentation (39d; *n* = 2,166). Blue edges indicate positive associations and red edges indicate negative associations. The width and saturation of the edge indicates strength of the relationship, with thicker and more saturated edges representing stronger associations. Networks are plotted by calculating the average layout of the networks, and then constraining each of these networks to that layout. Within each network, the colour of the node indicates its cluster membership as defined by the walktrap algorithm (covariates have been forced into their own category). For all binary nodes , the orange colour around each node indicates the accuracy achieved by the marginal (i.e., unadjusted model); the red colour around each node indicates the additional accuracy achieved by all nodes that are connected to that node. Red + orange denotes the accuracy of the full model (i.e., marginal + additional accuracy). Normalised accuracy is depicted by the ratio of red/(red + white). Normalised accuracy is the accuracy achieved by all nodes it is connected to, beyond the accuracy achieved by the marginal. For the continuous node (i.e., age), the blue bar indicates the explained variance by all nodes it is connected to.

**3.7 Diagnosis-level analysis: Eliminating purging-only cases**

**3.7.1 Centrality**

‘Irritability’ was the least central node in the bulimia nervosa and binge-eating disorder networks and ‘more energy’ was the most central. In the anorexia nervosa binge-eating network, ‘reckless spending’ was the least central and ‘hyperactivity’ the most central (Supplementary Figure 40). ‘More energy’ was significantly more central (*p* < 0.001) in the bulimia nervosa group than in the anorexia nervosa binge-eating group.

**
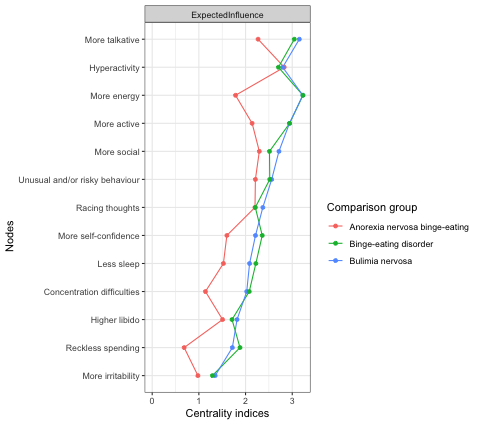
**

**SUPPLEMENTARY FIGURE 40** Centrality plot comparing the standardised expected influence of each symptom node in the groups within the diagnosis-level analysis, after eliminating purging-only cases (anorexia nervosa binge-eating *n* = 400; bulimia nervosa *n* = 3,830; binge-eating disorder *n* = 3,667).

**3.7.2 Node accuracy**

‘More energy’, ‘hyperactivity’, and ‘more talkative’ had the highest accuracy in the anorexia nervosa binge-eating network (0.70, 0.64, and 0.64), whilst reckless spending had the lowest (0.00; Supplementary Table 12). ‘More energy’ (0.72 and 0.70), and ‘more active’ (0.70 and 0.70) had the highest normalised accuracy in the bulimia nervosa and binge-eating disorder networks, and ‘irritability’ had the lowest normalised accuracy.

**SUPPLEMENTARY TABLE 12** Normalised accuracy of each node in networks of people with binge-type eating disorders (anorexia nervosa binge-eating *n* = 400; bulimia nervosa *n* = 3,830; binge-eating disorder *n* = 3,667) in the sensitivity analysis in which we eliminated participants with purging only in the anorexia nervosa binge-eating/purging group. Participants are from the National Institute for Health and Care Research (NIHR) BioResource. Diagnoses have been assigned using self-report (via the Mental Health Diagnosis [MHD] questionnaire) and/or diagnostic algorithms based on the Diagnostic and Statistical Manual of Mental Disorders, Fifth Edition (DSM-5; via the ED100K) in either the Genetic Links to Anxiety and Depression (GLAD) Study, the Eating Disorders Genetics Initiative (EDGI UK), and/or the COVID-19 Psychiatry and Neurological Genetics (COPING) study. Participants are a sub-sample of the people who report binge eating.

|  | **Normalised accuracy** | | |
| --- | --- | --- | --- |
|  | **Anorexia nervosa binge-eating** | **Bulimia nervosa** | **Binge-eating disorder** |
| Hyperactivity | 0.64 | 0.61 | 0.53 |
| Irritability | 0.25 | 0.09 | 0.08 |
| More self-confidence | 0.51 | 0.55 | 0.53 |
| Less sleep | 0.53 | 0.47 | 0.48 |
| More talkative | 0.64 | 0.57 | 0.58 |
| Racing thoughts | 0.48 | 0.28 | 0.27 |
| Concentration difficulties | 0.36 | 0.23 | 0.21 |
| More energy | 0.70 | 0.72 | 0.70 |
| More active | 0.59 | 0.70 | 0.70 |
| More social | 0.54 | 0.49 | 0.42 |
| Higher libido | 0.50 | 0.48 | 0.45 |
| Unusual and/or risky behaviour | 0.57 | 0.52 | 0.50 |
| Reckless spending | 0.00 | 0.29 | 0.32 |

**3.7.3 Network comparison**

Comparing the symptom networks of those with anorexia nervosa binge-eating (*n* = 400) to that of the bulimia nervosa (*n* = 3,830) and binge-eating disorder (*n* = 3,667) networks indicated no significant differences in global strength (S = 5.59 and 5.25, *p*s > 0.3). The network structure of anorexia nervosa binge-eating also did not differ significantly from the binge-eating disorder network (M = 0.52, *p* = 0.06), however it did differ significantly to the bulimia nervosa network (M = 0.74, *p* = 0.002) (Supplementary Figure 41). The edge weight between ‘racing thoughts’ and ‘more talkative’ was significantly stronger in the bulimia nervosa network (0.74) than the anorexia nervosa binge-eating network (0, *p* < 0.001).

**
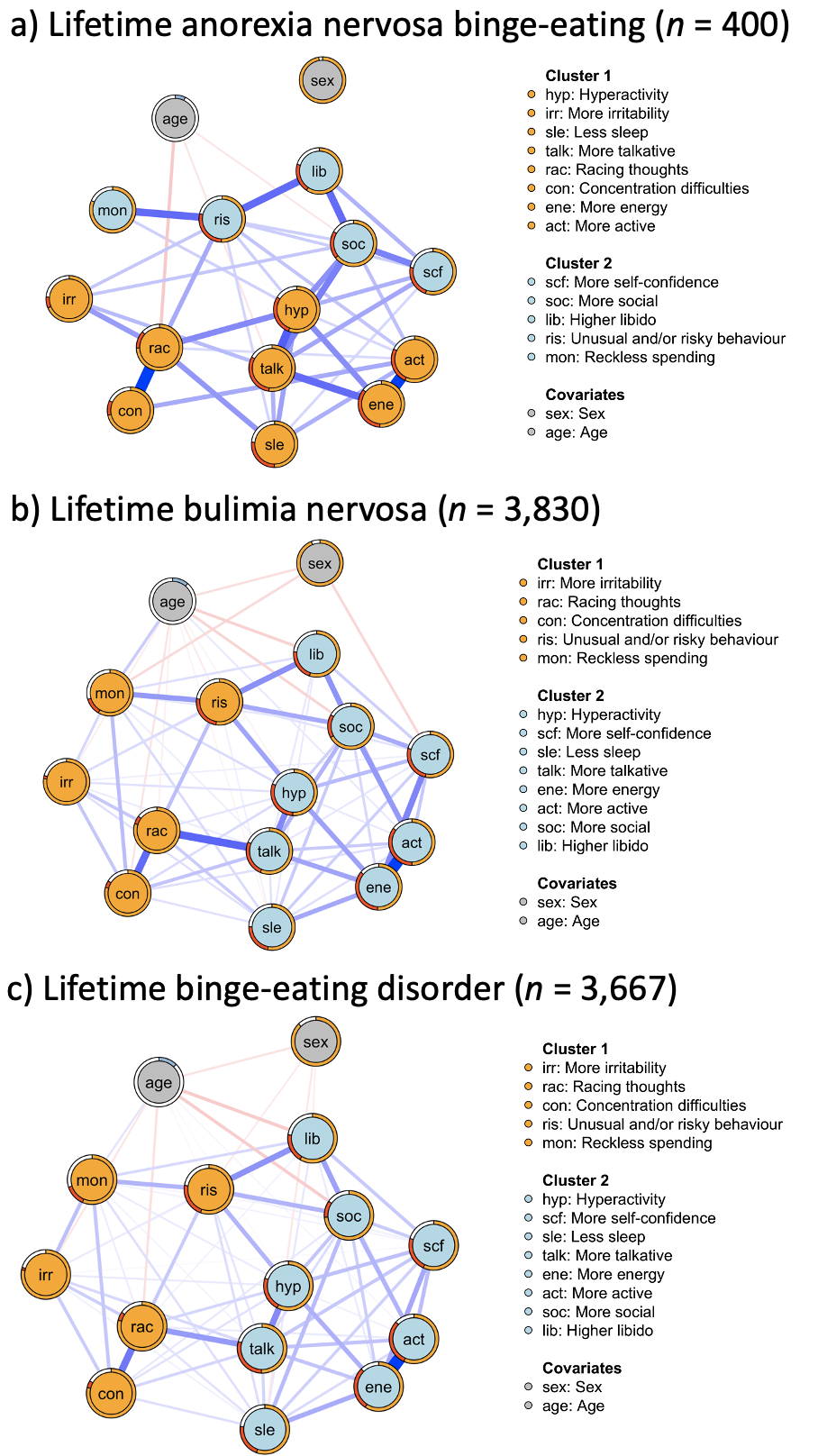
**

**SUPPLEMENTARY FIGURE 41** Mania symptom networks in individuals hierarchically categorised into groups of lifetime diagnosis of anorexia nervosa binge-eating (41a; *n* = 400), bulimia nervosa (41b; *n* = 3,830), binge-eating disorder (41c; *n* = 3,667). Blue edges indicate positive associations and red edges indicate negative associations. The width and saturation of the edge indicates strength of the relationship, with thicker and more saturated edges representing stronger associations. Networks are plotted by calculating the average layout of the networks, and then constraining each of these networks to that layout. Within each network, the colour of the node indicates its cluster membership as defined by the walktrap algorithm (covariates have been forced into their own category). For all binary nodes , the orange colour around each node indicates the accuracy achieved by the marginal (i.e., unadjusted model); the red colour around each node indicates the additional accuracy achieved by all nodes that are connected to that node. Red + orange denotes the accuracy of the full model (i.e., marginal + additional accuracy). Normalised accuracy is depicted by the ratio of red/(red + white). Normalised accuracy is the accuracy achieved by all nodes it is connected to, beyond the accuracy achieved by the marginal. For the continuous node (i.e., age), the blue bar indicates the explained variance by all nodes it is connected to.

**References**

1. [Hirschfeld RM, Williams JB, Spitzer RL, Calabrese JR, Flynn L, Keck PE Jr, et al. Development and validation of a screening instrument for bipolar spectrum disorder: the Mood Disorder Questionnaire. Am J Psychiatry. 2000;157: 1873–1875.](http://paperpile.com/b/sZAmz0/gd6Yp)

2. [Epskamp S, Borsboom D, Fried EI. Estimating psychological networks and their accuracy: A tutorial paper. Behav Res Methods. 2018;50: 195–212.](http://paperpile.com/b/sZAmz0/Su8nU)

3. [Haslbeck JMB, Waldorp LJ. mgm: Estimating Time-Varying Mixed Graphical Models in High-Dimensional Data. arXiv [stat.AP]. 2015. Available:](http://paperpile.com/b/sZAmz0/qOU1d) <http://arxiv.org/abs/1510.06871>

4. [McNally RJ. Can network analysis transform psychopathology? Behav Res Ther. 2016;86: 95–104.](http://paperpile.com/b/sZAmz0/NZUfj)

5. van Borkulo CD, Boschloo L, Kossakowski J, Tio P, Schoevers RA, Borsboom D, et al. Comparing network structures on three aspects: A permutation test. Psychol Methods. 2022.

6. [Fried EI, van Borkulo CD, Cramer AOJ, Boschloo L, Schoevers RA, Borsboom D. Mental disorders as networks of problems: a review of recent insights. Soc Psychiatry Psychiatr Epidemiol. 2017;52: 1–10.](http://paperpile.com/b/sZAmz0/CODTP)

7. [Bringmann LF, Elmer T, Epskamp S, Krause RW, Schoch D, Wichers M, et al. What do centrality measures measure in psychological networks? J Abnorm Psychol. 2019;128: 892–903.](http://paperpile.com/b/sZAmz0/WcFiG)

8. [Epskamp S, Fried EI. A tutorial on regularized partial correlation networks. Psychol Methods. 2018;23: 617–634.](http://paperpile.com/b/sZAmz0/xKnh3)

9. [Haslbeck JMB, Waldorp LJ. How well do network models predict observations? On the importance of predictability in network models. Behav Res Methods. 2018;50: 853–861.](http://paperpile.com/b/sZAmz0/Nt6I)
